# Cytological studies reveal high variation in ascospore number and shape and conidia produced directly from ascospores in *Morchella galilaea*

**DOI:** 10.3389/fmicb.2023.1286501

**Published:** 2023-11-09

**Authors:** Xi-Hui Du, Si-Yue Wang, Martin Ryberg, Yong-Jie Guo, Jing-Yi Wei, Donald H. Pfister, Hanna Johannesson

**Affiliations:** ^1^College of Life Sciences, Chongqing Normal University, Chongqing, China; ^2^Evolution Biology Centre, Department of Organismal Biology, Uppsala University, Uppsala, Sweden; ^3^University of Chinese Academy of Sciences, Beijing, China; ^4^Germplasm Bank of Wild Species, Kunming Institute of Botany, Chinese Academy of Sciences, Kunming, China; ^5^Chongqing Academy of Agricultural Sciences, Chongqing, China; ^6^Farlow Reference Library and Herbarium and Department of Organismic and Evolutionary Biology, Harvard University, Cambridge, MA, United States; ^7^Department of Ecology, Environment and Plant Sciences, Stockholm University, Stockholm, Sweden; ^8^The Royal Swedish Academy of Sciences, Stockholm, Sweden

**Keywords:** aborted ascospore, ascomycetes, ascospore number, ascospore shape, budding, ascoconidium, meiosis, mitosis

## Abstract

Spores are important as dispersal and survival propagules in fungi. In this study we investigated the variation in number, shape, size and germination mode of ascospores in *Morchella galilaea*, the only species of the genus *Morchella* known to fruit in the autumn. Based on the observation of five samples, we first discovered significant variation in the shape and size of ascospores in *Morchella*. One to sixteen ascospores were found in the asci. Ascospore size correlated negatively with ascospore number, but positively with ascus size, and ascus size was positively correlated with ascospore number. We noted that ascospores, both from fresh collections and dried specimens, germinated terminally or laterally either by extended germ tubes, or via the production of conidia that were formed directly from ascospores at one, two or multiple sites. The direct formation of conidia from ascospores takes place within asci or after ascospores are discharged. Using laser confocal microscopy, we recorded the number of nuclei in ascospores and in conidia produced from ascospores. In most ascospores of *M. galilaea*, several nuclei were observed, as is typical of species of *Morchella*. However, nuclear number varied from zero to around 20 in this species, and larger ascospores harbored more nuclei. One to six nuclei were present in the conidia. Nuclear migration from ascospores to conidia was observed. Conidia forming directly from ascospores has been observed in few species of Pezizomycetes; this is the first report of the phenomenon in *Morchella* species. Morphological and molecular data show that conidial formation from ascospores is not found in all the specimens of this species and, hence, is not an informative taxonomic character in *M. galilaea*. Our data suggest that conidia produced from ascospores and successive mitosis within the ascus may contribute to asci with more than eight spores. The absence of mitosis and/or nuclear degeneration, as well as cytokinesis defect, likely results in asci with fewer than eight ascospores. This study provides new insights into the poorly understood life cycle of *Morchella* species and more broadly improves knowledge of conidia formation and reproductive strategies in Pezizomycetes.

## 1. Introduction

Ascomycetes produce two kinds of spores in order to propagate and colonize. Ascospores are produced after meiosis and conidia are produced by mitosis (Pöggeler et al., 2006; Nieuwenhuis and James, 2016). In addition to providing novel genetic variants by recombination during meiosis, ascospores are important both for long-distance dispersal and resistance to harsh environments (Chaudhary et al., 2022). One ascus generally produces eight ascospores (Pöggeler et al., 2006), but the number of ascospores within an ascus varies between one and > l000 depending on the species (Braus et al., 2002). Variation in ascospore number is likely determined by the coordination of meiosis, mitosis and spore wall formation, and may be driven by natural selection (Davidow and Goetsch, 1978; Sherwood, 1981; Réblová and Mostert, 2007). For example, an increased ascospore number leads to a higher number of propagules for dispersal and colonization, and four-spored asci may contain heterokaryotic multinucleate and self-fertile ascospores, as in pseudohomothallic species (Hanlin, 1994; Raju and Perkins, 1994; Quijada et al., 2022). Investigating the variation in ascospore size and number in asci helps to understand the cytology, genetics and life cycle of fungi.

The conidium is the main type of mitospores produced by ascomycetes and serves as a propagule for rapid dissemination or as a “safe house” for the fungal genomes under adverse environmental conditions (Goh et al., 2009). Typically, after a period of vegetative growth conidia are produced on conidiophores, which are differentiated from aerial hyphae (Adams et al., 1998). However, in certain ascomycete species, conidia are formed directly from the ascospores within asci of fresh and/or dried specimens, or after the ascospores are ejected. This phenomenon is uncommon in ascomycetes and has occasionally been described in several classes: Saccharomycetes from Saccharomycotina; Neolectomycetes and Taphrinomycetes from the Taphrinomycotina; and Lecanoromycetes, Leotiomycetes, Pezizomycetes and Sordariomycetes from the Pezizomycotina (Seaver, 1942; Juzwik and Hinds, 1984; Hawksworth et al., 1995; Ramaley, 1997; Baral, 1999; Wang et al., 2002; Ertz and Diederich, 2004; Neiman, 2005; Frisch and Klaus, 2006; Réblová and Mostert, 2007; Hirooka et al., 2012; Quijada, 2015; Réblová et al., 2015; Zeng and Zhuang, 2016; Lechat et al., 2018; Réblová and Štěpánek, 2018; Quijada et al., 2019; Van Vooren, 2020; Huang et al., 2021; Karakehian et al., 2021).

The phenomenon of formation of conidia directly from ascospores, without mycelial growth, can be termed microcyclic conidiation (Vinter and Slepecky, 1965; Hawksworth 1987; Hanlin, 1994; Baral, 1999; Quijada, 2015; Quijada et al., 2019). However, we note that Baral (1999) defined the term “ascoconidia” strictly as conidia produced from ascospores contained within living asci (i.e., fresh specimens), in which each ascospore together with its ascoconidia are surrounded by a delicate membrane forming a ball that is violently ejected from asci as a single entity. In contrast, conidia formed from ejected ascospores or within dead asci (i.e., dried specimens), which are not arranged as balls, are simply referred to as conidia. Such conidia or ascoconidia have been suggested to be beneficially associated with drought-tolerance (Sherwood, 1981), colonization of new habitats (Quijada et al., 2022), infection of new hosts in harsh environments (Juzwik and Hinds, 1984; Hanlin, 1994) and to serve as spermatia (Hanlin, 1994). Observations of ascospore germination and conidial formation provide important information on development in fungi (Karakehian et al., 2021).

In the Pezizomycetes, ascoconidia, as defined by Baral (1999), are not found, but conidial formation directly from ascospores has been rarely reported; it is mentioned in *Purpureodiscus subisabellinus* (Van Vooren, 2020) and a few other examples. It has not been previously reported in the Morchellaceae. True morels (*Morchella* spp.) belonging to Morchellaceae are well-known as one of the most popular edible fungi in the world, due to their highly desirable flavor (Du et al., 2012). In this genus, diverse reproductive modes have been reported, i.e., heterothallism, pseudohomothallism or unisexual reproduction, and asexually by formation of conidia (Chai et al., 2017, 2019; Du et al., 2017, 2020; Du and Yang, 2021). *Costantinella* was previously described as the conidial or mitosporic morph of *Morchella* species (Molliard, 1904) but following the elimination of dual naming systems for teleomorphs and anamorphs, *Morchella* has been recommended as the generic name (Healy et al., 2016). The *Costantinella* morph produces conidia on erect conidiophores and has been discussed recently by Carris et al. (2015); Liu et al. (2016); Yuan et al. (2021); Pfister et al. (2022). Considering the diverse modes of reproduction in *Morchella*, studies on ascospore morphology, ascospore germination and conidial formation may help elucidate the complex life cycles of these species.

*Morchella galilaea* is distributed globally. It is the only species in the genus *Morchella* known to produce ascomata in autumn in the Northern Hemisphere (Du et al., 2015; Taşkin et al., 2015). This temporal difference in ascomatal production, compared with the other species of the genus, may reflect potential special climate and ecological preferences and impart ecological advantages for *M. galilaea*.

The objectives of the current study are to investigate the cytology and variation in shape, number, and size of ascospores and asci in *M. galilaea*. We revealed a considerable variation in ascospore shape and number per ascus and clear correlations between ascospore size, ascospore number and ascus size. Furthermore, we discovered that conidia are produced directly from ascospores within asci and from discharged ascospores of both fresh and dried specimens. We observed nuclear distribution and migration from ascospores to conidia and determined the number of nuclei within these spores. More broadly we compare these features among the Pezizomycetes. Our results further add to the knowledge of ascospore development and types of conidial formation in *Morchella* species.

## 2. Materials and methods

### 2.1. Specimen isolation and storage

**Figure 1:**
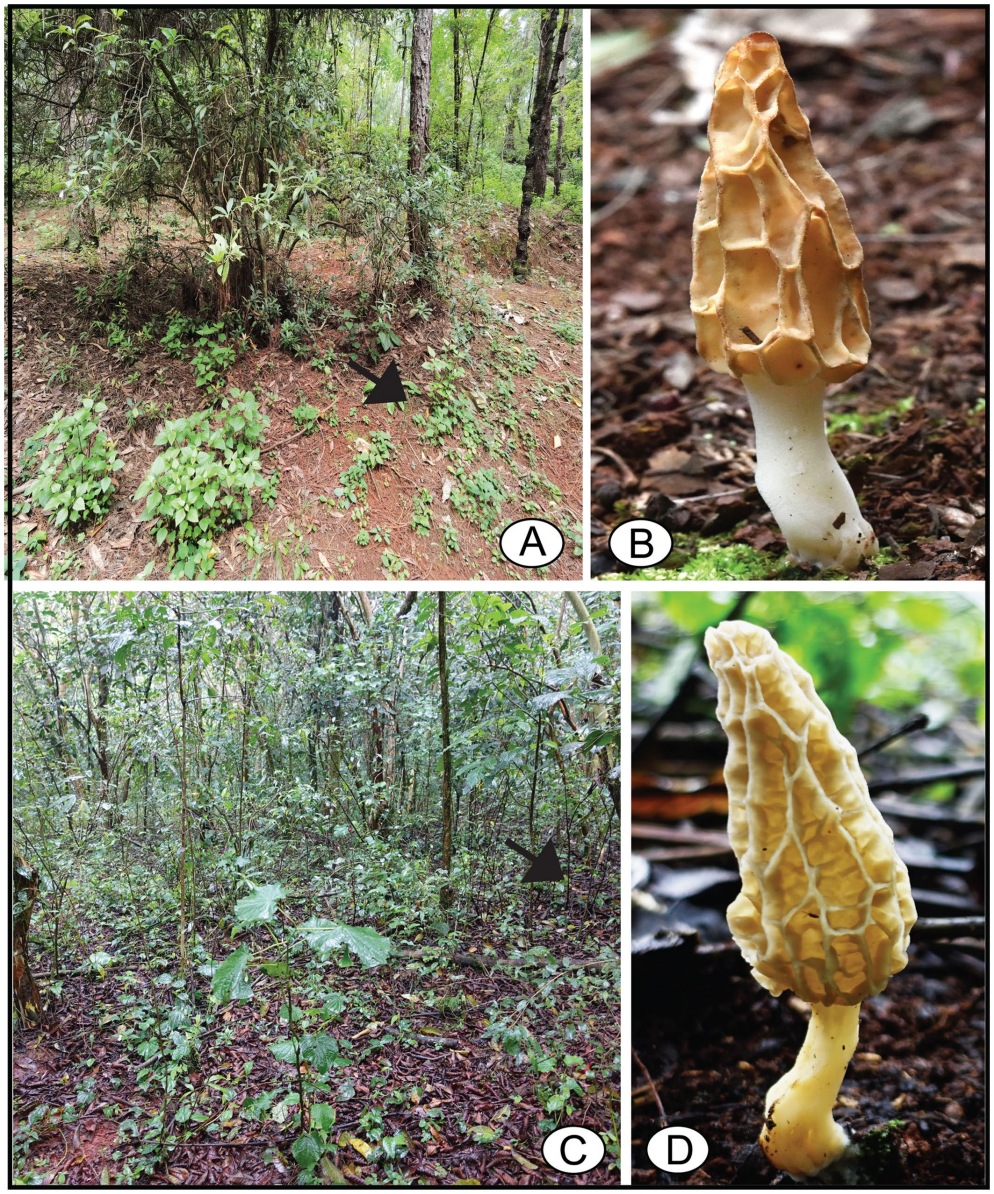
Habitat and ascomata of *Morchella galilaea*. **(A,B)**: FCNU1119 from China; **(C,D)**: FCNU1061 from Kenya. Ascomata indicated by arrows in their habitat.

Five ascomata of *M. galilaea*, collected in China and Kenya from 2015 to 2022, were used as study materials. Among the five specimens, four were dried and one was freshly collected in 2022. The ascus state is presumed to be living for the fresh specimen and dead for dried specimens. All the samples were dried with silica gel and deposited in Chongqing Normal University, Chongqing, China. The habitat and ascomata of two specimens are shown in Figure 1 and details of all the specimens are listed in Table 1.

**Table 1:** Voucher information, GenBank accession numbers, conidial production, ascospore shapes and ascospore number per ascus of the five samples of *Morchella galilaea* used in this study.

Voucher	Locality	Collection date	Specimen state	GenBank accession number				Conidia produced in asci	Ascospore shapes (No. of elliptical spores/ No. of total spores observed)	Ascospore numbers per ascus
				ITS	*EF1a*	*RPB1*	RPB2			
FCNU1061	Kakamega Forest, Kenya	2015.12	Herbarium specimen (✝)	MN513736	MN513663	OR497003	OR497008	No	Elliptical, spherical, crescent (152/156)	5, 8, 9, 10
FCNU1116	Sichuan, China	2017.08	Herbarium specimen (✝)	OR501830	OR496996	OR497000	OR497005	Yes	Elliptical, spherical, heart-shaped, crescent, etc. (162/169)	3, 4, 5, 6, 7, 8, 9
FCNU1117	Sichuan, China	2020.10	Herbarium specimen (✝)	OR501832	OR496998	OR497002	OR497007	Yes	Elliptical, spherical, heart-shaped, crescent, etc. (168/176)	1, 2, 6, 7, 8, 9, 10, 11
FCNU1118	Sichuan, China	2020.10	Herbarium specimen (✝)	OR501831	OR496997	OR497001	OR497006	Yes	Elliptical, spherical, heart-shaped, crescent, etc. (175/182)	1, 2, 3, 4, 5, 6, 7, 8, 9, 10, 11, 12, 13, 14, 15, 16
FCNU1119	Yunnan, China	2022.10	Fresh collection (✻)	OR501829	OR496995	OR496999	OR497004	Yes	Elliptical, spherical, heart-shaped, crescent, etc. (155/162)	2, 5, 6, 7, 8, 9

✝ = dried specimen; ✻ = fresh specimen.

### 2.2. DNA extraction, sequencing, and phylogenetic analyses

The specimens were initially identified as *M. galilaea* based on their autumnal occurrence. Further molecular phylogenetic analyses were conducted to confirm the species’ identity. Methods for genomic DNA extraction, PCR amplification and Sanger sequencing followed those described by Du et al. (2012). The following primer pairs were used for PCR amplification and sequencing of four markers: ITS1/ITS1F and ITS4 for the internal transcribed spacers 1 and 2 within 5.8S rDNA (ITS) (White et al., 1990; Gardes and Bruns, 1993); EF595F and EF2218R/EF1R for the translation elongation factor 1-a (*EF1-a*) (Kauserud and Schumacher, 2001; Rehner and Buckley, 2005; Du et al., 2012); RPB1Y-F and RPB1Y-R for the RNA polymerase I second largest subunit (*RPB1*) (Du et al., 2012); and, RPB2Y-F and RPB2Y-R for the RNA polymerase II second largest subunit (*RPB2*) (Du et al., 2012). The amplicons were sequenced with the ABI 3730 capillary sequencer (Applied Biosystems, Foster City, CA). Newly generated sequences were assembled and edited using SeqMan (DNA STAR package; DNAStar Inc., Madison, WI, United States). In addition, 120 sequences of ITS, *EF1-a*, *RPB1* and *RPB2* markers from thirteen species in the Esculenta clade, namely *M. americana*, *M. esculenta*, *M. galilaea*, *M. gracilis*, *M. prava*, *M. sceptriformis*, *M. steppicola*, *M. ulmaria*, *Morchella* sp. *Mes*-6, *Morchella* sp. *Mes*-9, *Morchella* sp. *Mes*-15, *Morchella* sp. *Mes-*25 and *Morchella* sp. *Mes*-26 (Taşkın et al., 2010; O’Donnell et al., 2011; Du et al., 2012; Taşkın et al., 2012) were retrieved from GenBank and included in the analyses. Accession numbers of these retrieved sequences are given in Supplementary Table S1.

Newly generated sequences were combined in an alignment with the 120 sequences of thirteen representative species of the Esculenta clade. Sequence alignments were performed separately for each marker with MAFFT v6.853 using the E-INS-i strategy (Katoh et al., 2002), manually checked with BioEdit 7.0.9 (Hall, 1999), and then concatenated using SequenceMatrix v1.7.8 (Vaidya et al., 2011). Both maximum likelihood (ML) and Bayesian inference (BI) phylogenetic analyses were used to analyze the combined four-marker dataset (ITS-*EF1a*-*RPB1*-*RPB2*) by using RAxML v.8.2.4 (Stamatakis, 2014) and MrBayes v.3.2 (Ronquist et al., 2012), respectively. *Morchella steppicola*, which is the basal species in the Esculenta clade (O’Donnell et al., 2011; Du et al., 2015), was chosen as the outgroup. The partitioned analysis model was employed in the phylogenetic analysis of the combined four-marker dataset. A rapid bootstrapping with 1,000 replicates was executed with the GTR + GAMMA+I model used in ML analysis. The BI analysis used four Markov Chain Monte Carlo (MCMC) chains and was run for one million generations, with trees sampled every 100 generations. Runs were automatically terminated when the average standard deviation of split frequencies fell below 0.01. The trees were summarized with burn-ins of the first 25% of samples using the “sump” and “sumt” commands to obtain posterior possibilities.

### 2.3. Morphological studies

Morphological descriptions of asci, ascospores and conidia were based on observations of all five samples available. Hand sections for microscopic examination were prepared with a safety razor blade. Potassium hydroxide (5%) was used to rehydrate dried specimens prior to morphological analysis. Specimens were stained with 1% aqueous Congo red solution when necessary. Microscopic features were observed using an Optec BK-FL light microscope (Optec, Chongqing, China) at magnifications of 40×, 100×, 400×, and 1,000×, and were drawn by hand. Images were captured with an Optec CCD TP510 digital camera (Optec). Measurements of ascospores are presented with 95% confidence intervals. Ascospore sizes are presented using a range notation in the form (a-) b-c (−d), where the range b-c contains a minimum of 90% of the measured values and extreme values (a, d) are shown in parentheses.

Nuclei were stained with 4′,6-diamidino-2-phenilindole (DAPI; Sigma-Aldrich, St Louis MO, United States) for 10 min directly on microscope slides. Excess stain was removed with filter paper. Nuclei were visualized with a Fluorview 1,000 laser confocal fluorescent microscope (Olympus, Tokyo, Japan). Images were analyzed using FV10-ASW 4.0 Viewer software (Olympus). The descriptions and abbreviations follow those of Baral (1999): * = living state; † = dead state. Images were assembled and processed using Adobe Photoshop CC 2017 or Illustrator CC (Adobe Systems, San Jose, CA). Correlation analyses were performed to evaluate the relationship among ascospore number, ascospore size and ascus size. Data were plotted on scatter diagrams.

## 3. Results and discussion

### 3.1. Molecular phylogenetic analysis of *M*orchella *galilaea* specimens

**Figure 2:**
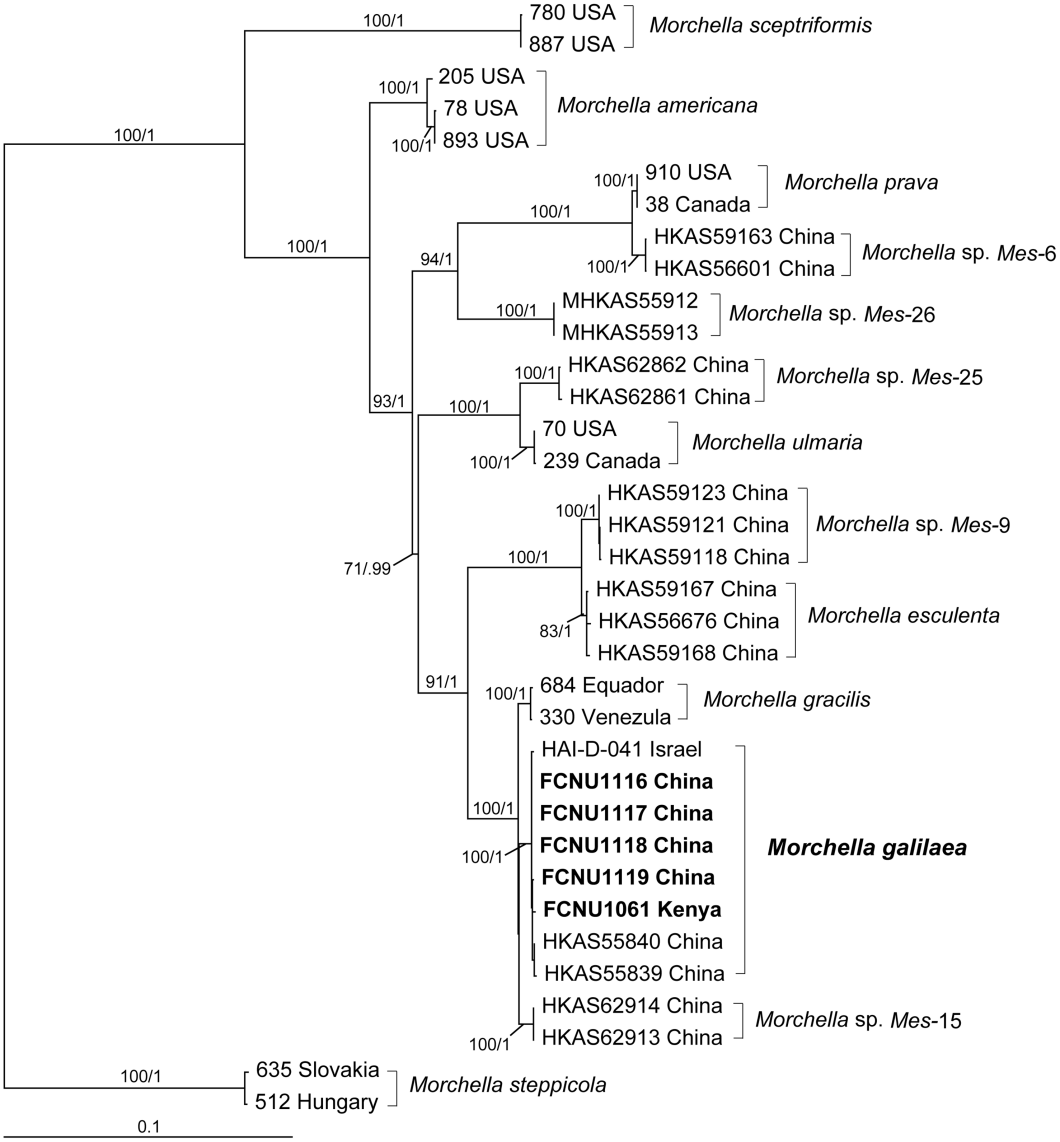
Phylogenetic tree of thirteen species in the Esculenta clade of *Morchella* inferred from ML analyses based on the concatenated dataset (ITS, *EF1-α*, *RPB1*, and *RPB2*). Bootstrap values over 70% and Bayesian posterior probabilities over 0.95 are reported on the branches. Sequences generated from collections of *Morchella galilaea* used in this study are indicated in bold.

By using molecular phylogenetic analyses, we verified the species identity of the specimen used in this study as *M. galilaea*. The alignments of the twenty sequences of four markers that were newly generated in this study (Table 1) and the 120 retrieved sequences from GenBank (Supplementary Table S1) were 1,096, 895, 716, and 729 bp, for ITS, *EF1-a*, *RPB1*, and *RPB2*, respectively. The final aligned multi-marker matrix contained 35 collections belonging to 13 species, and total 140 sequences with 3,436 aligned bases. The phylogenetic trees were inferred from the combined four-marker dataset based on ML and BI analyses. No apparent topological differences were detected between the two analyses (data not shown), and the ML phylogeny is presented in Figure 2. The phylogenetic analyses strongly supported the studied specimens as *M. galilaea* (Figure 2) since they were clustered together with HKAS55840 and HKAS55839 from China and HAI-D-041 from Israel with high support (100%/1); these were previously identified as *M. galilaea* (Du et al., 2012; Taşkin et al., 2015). Among the five samples studied, those from Kenya showed three unique single base pair variants relative to the specimens from China, but clustered closely with them with high bootstrap support (100%/1), suggesting low geographic differentiation.

### 3.2. Morphological studies of *M*orchella *galilaea* specimens

We identified considerable morphological variation in ascospore shape, number of ascospores within asci, conidial formation from ascospores and nuclear number in ascospores and conidia from the specimens of *M. galilaea*.

#### 3.3.1. Ascospore shape and size are variable in *M*orchella *galilaea*

**Figure 3:**
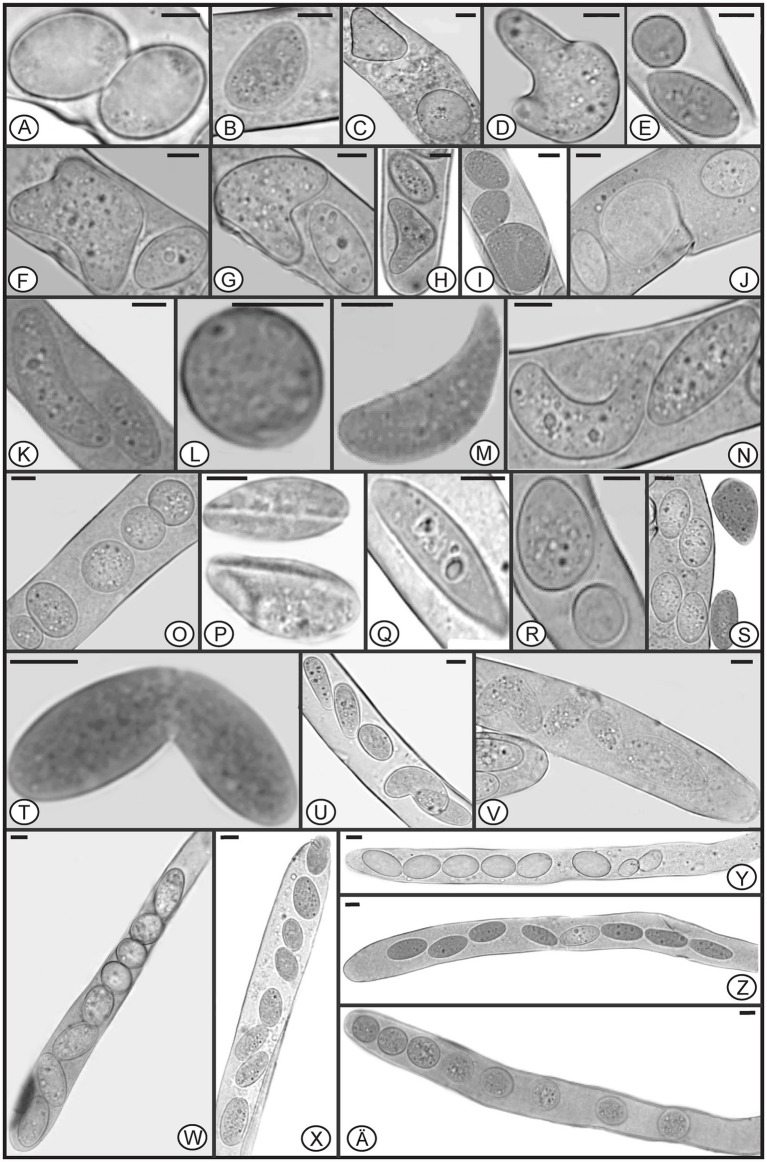
Diverse shapes of ascospores within or outside asci of *Morchella galilaea* **(A-Z, Ä)**. Scale bars = 5 μm.

**Figure 4:**
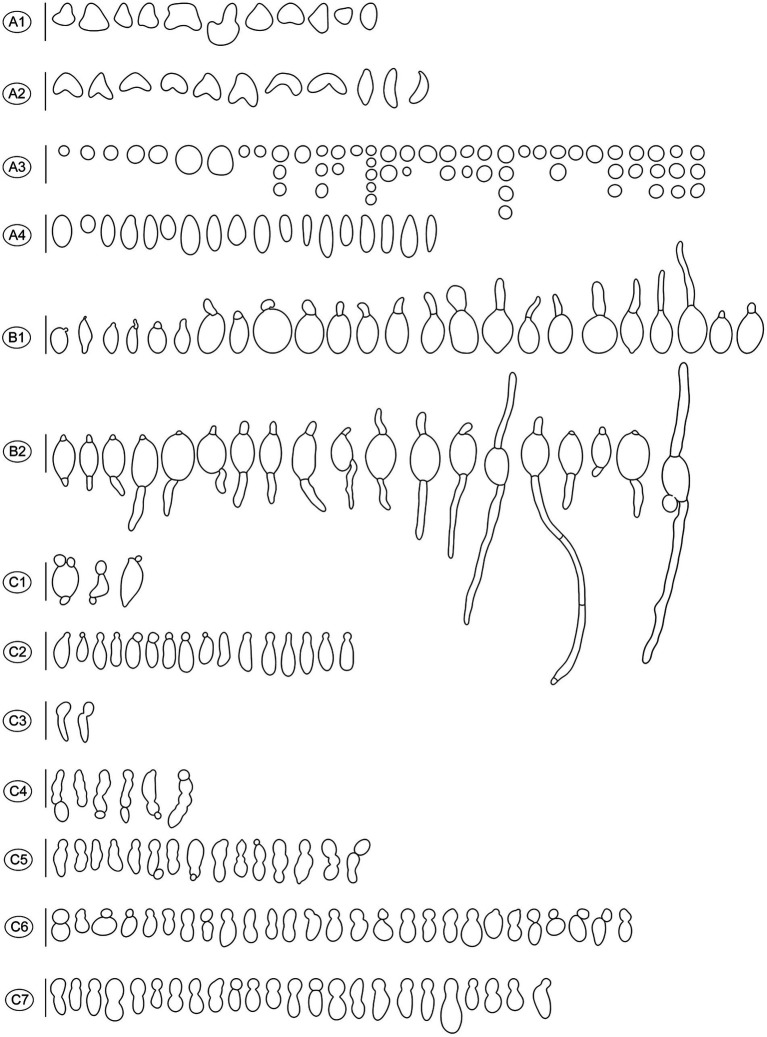
Diverse shapes of ascospores and germination either by germ tube or via conidia formation in *Morchella galilaea* drawn following observation by microscopy. **(A)**: diverse shape of ascospores and detached conidia (small globose conidia in A3); **(B)**: ascospore germination by the long germ tube or via conidia production; **(C)**: conidia from ascospores in single or chains. Scale bars = 20 μm.

In *Morchella* species, mature ascospores are typically elliptical (Clowez et al., 2014, 2015; Taşkin et al., 2015; Loizides et al., 2016; Du et al., 2019), however, various ascospore shapes were observed among the specimens of *M. galilaea* (Figure 3). Elliptical ascospores dominated and accounted for 96.1% (total 845 spores investigated from the five samples, detailed information shown in Table 1), but irregular shapes, including spherical, heart-shaped, crescent, and other forms were also observed in all investigated fruiting bodies (Table 1, Figure 3). In Figure 4, the diversity of ascospore shapes and sizes is illustrated using the same scale for each element. Diversity of ascospore shape was not only found in different asci from the five samples (Table 1), but also from the same ascus (Figures 3W–Z,A). Certain asci harbored spores that were almost all spherical (Figure 3Ä) but several different spore shapes also were evident within a single ascus (Figures 3W–Z). The size of typically elliptical ascospores was (11.7-)16.8–22.9(−25.5) × (8.0-)9.6–14.5(−16.1) μm (average Q = 1.66) based on data from 200 ascospores of the five specimens (40 from each), whereas differently shaped spores were (7.9-)10.0–24.2(−29.1) × (5.8-)6.8–13.4(−19.6) μm (average Q = 1.82) based on observation of 236 spores from five specimens (13/ FCNU1061, 13/FCNU1116, 13/FCNU1117, 183/FCNU1118 and 14/FCNU1119).

Ascospore shape has been suggested to correlate with ecological niche and was shown to have an influence on the efficiency of active spore discharge (Ingold, 1975; Raju and Burk, 2004; Campbell et al., 2006; Shenoy et al., 2006; Hirooka et al., 2012). Most explosively ejected spores in ascomycetes have a drag-minimizing ellipsoid shape close to the theoretical optimum, which maximizes the range of their active liberation (Roper et al., 2008). Successful liberation of spores from ascomata facilitates dispersal. The irregular ascospore morphologies observed, such as spherical and heart shapes, seem to present difficulties for ascospore discharge compared to elliptical shapes. It is unknown whether these variant ascospore shapes are aberrations or have some unidentified function. The formation of moon– and heart-shaped ascospores in *M. galilaea* appears to arise from incomplete spore delimitation between two spores. Whether these variant spore shapes have to do with the autumn fruiting of *M. galilaea* remains to be studied. In further study, we will focus on whether these variant ascospores germinate and try to discover whether there is a potential function associated with these shapes.

#### 3.3.2. Variation in ascospore number in *M*orchella *galilaea*

**Figure 5:**
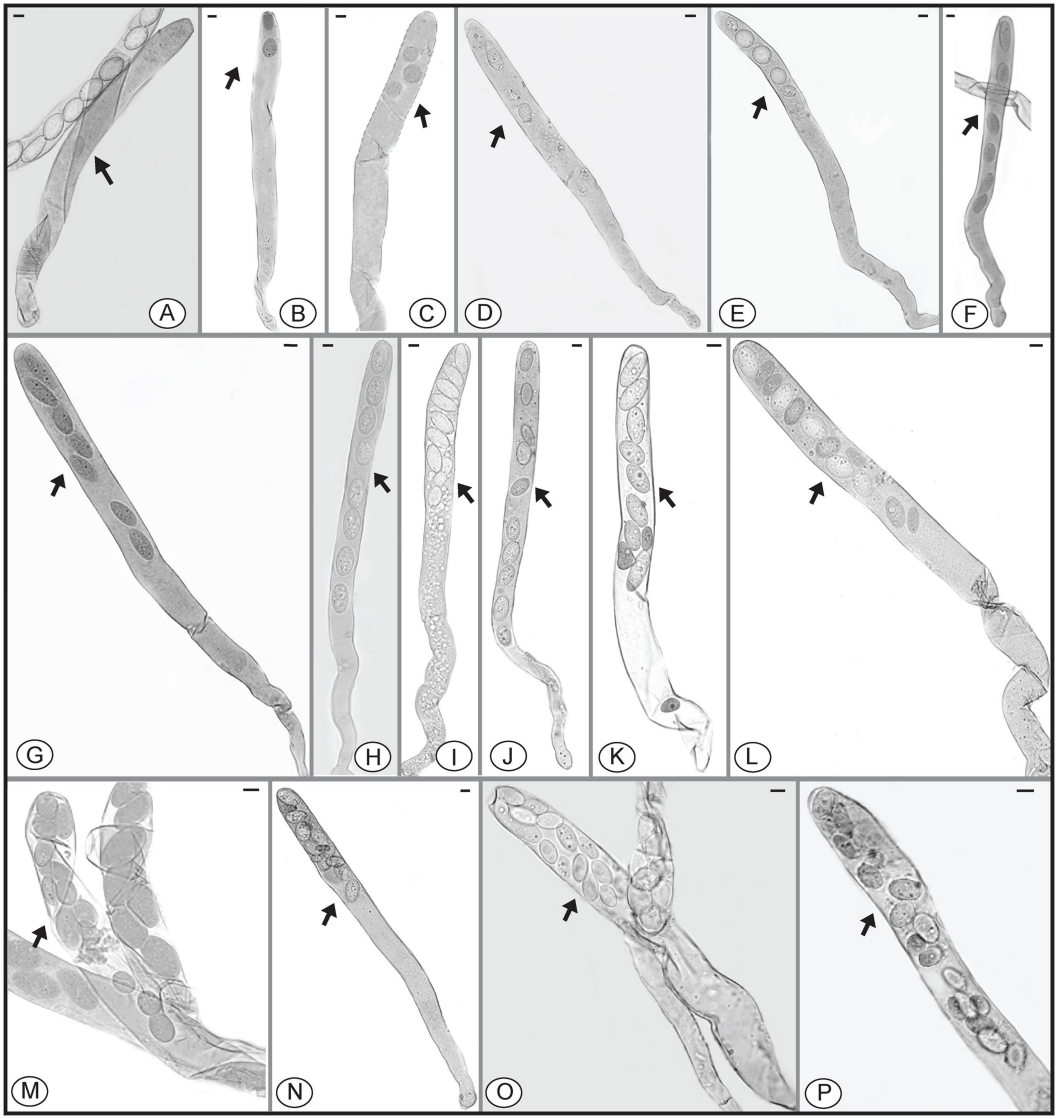
Asci of *Morchella galilaea* harboring one to 16 ascospores. **(A)**: one-spored ascus; **(B)**: two-spored ascus; **(C)**: three-spored ascus; **(D)**: four-spored ascus; **(E)**: five-spored ascus; **(F)**: six-spored ascus; **(G)**: seven-spored ascus; **(H)**: eight-spored ascus; **(I)**: nine-spored ascus; **(J)**: ten-spored ascus; **(K)**: 11-spored ascus; **(L)**: 12-spored ascus; **(M)**: 13-spored ascus; **(N)**: 14-spored ascus; **(O)**: 15-spored ascus; **(P)**: 16-spored ascus. Scale bars = 20 μm.

**Figure 6:**
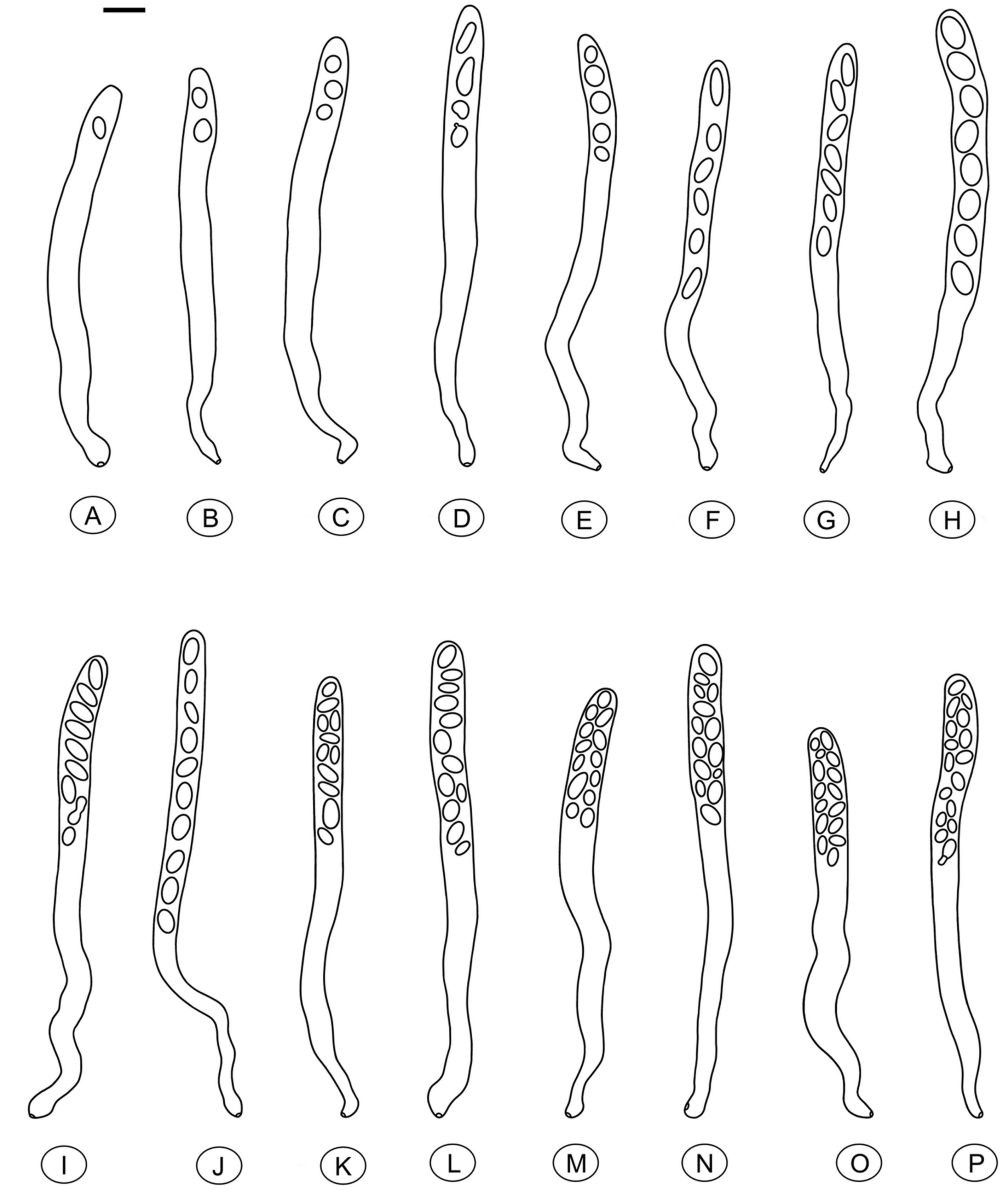
Asci of *Morchella galilaea* drawn following microscopic observation with conidia formation by budding from ascospores and diverse ascospore shapes shown. **(A)**: one-spored ascus; **(B)**: two-spored ascus; **(C)**: three-spored ascus; **(D)**: four-spored ascus; **(E)**: five-spored ascus; **(F)**: six-spored ascus; (G): seven-spored ascus; **(H)**: eight-spored ascus; **(I)**: nine-spored ascus; **(J)**: ten-spored ascus; **(K)**: 11-spored ascus; **(L)**: 12-spored ascus; **(M)**: 13-spored ascus; **(N)**: 14-spored ascus; **(O)**: 15-spored ascus; **(P)**: 16-spored ascus. Scale bar = 20 μm.

Species of *Morchella* have previously been shown to harbor eight ascospores per ascus (Clowez et al., 2014, 2015; Taşkin et al., 2015; Loizides et al., 2016; Du et al., 2019), but we found that the number of ascospores produced in asci of *M. galilaea* varied from one to 16 (Figure 5). The number of ascospores produced in asci studied are shown in Table 1. Mature ascus length varied from 215.6 μm (ascus with seven ascospores) to 364.4 μm (ascus with two ascospores), and the width ranged from 16.5 μm (ascus with two ascospores) to 23.87 μm (ascus with eight ascospores) (Supplementary Table S2). The ascus in this study ranged from 215.6–364.4 × 16.5–23.9 μm. This was greater than previously determined for *M. galilaea*, namely 165–220 × 15–22 μm (Taşkin et al., 2015). This ascus size difference may be attributed to the higher number of ascospores that were identified in this study (see below in 3.2.3). Asci and ascospores illustrated using the same scale make clear the variations of shape and size for the sixteen types observed (Figure 6).

**Figure 7:**
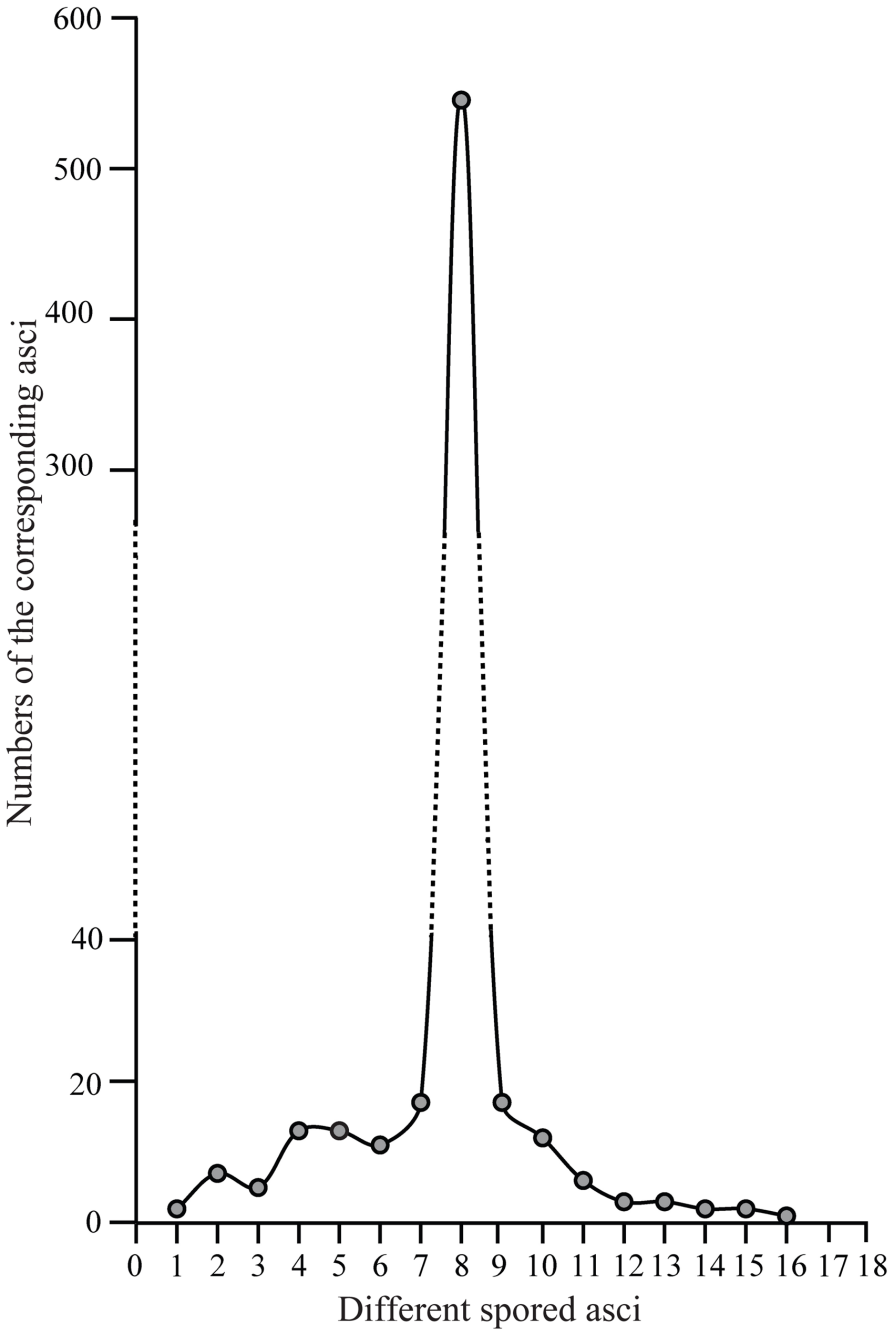
The distribution pattern of asci with one to 16 ascospores in *Morchella galilaea* according to analyses of 654 asci.

We further counted the number of asci with different numbers of ascospores (Figure 7, Supplementary Table S3). Their distribution pattern showed that asci with eight ascospores were predominant and accounted for 540/654 (82.6%) of the asci, which is consistent with the typical number of ascospores for most ascomycetes. Seven-spored and nine-spored asci were the second most numerous and were observed equally in 17/654 (2.6%) of asci. Asci that harbored 16 ascospores occurred least frequently (1/654; 0.15%). The second lowest prevalence was asci having one, 14, or 15 ascospores which each accounted for 2/654 (0.3%) of the total. Asci with other numbers of ascospores comprised 11.2% of the total (Supplementary Table S3).

In ascomycetes, species with varying numbers of spores per ascus were previously reported (Yao and Spooner, 2000; Bonito et al., 2010; Kušan et al., 2018; Pfister and Healy, 2021). However, variation in spore number within asci from the same species and from the same fruiting body are more rarely reported. Variation in spore number in yeast asci was suggested to depend closely on the carbon-to-nitrogen ratio of the growth medium, with more spores produced in media with a high carbon-to-nitrogen ratio, and fewer spores with a low ratio (Okuda, 1961). However, the carbon-to-nitrogen ratio is unlikely to be a factor in this study since spore numbers varied not only among different fruiting bodies, but also within a single fruiting body of *M. galilaea*. Variation in ascospore number has been suggested to be related to the lack of coordination of meiotic divisions, spore wall formation and successful mitotic divisions. Varying propagule number may contribute to differences in dispersal range, colonization of new habitats, or the production of heterokaryotic ascospores for pseudohomothallic species (Davidow and Goetsch, 1978; Sherwood, 1981; Hanlin, 1994; Raju and Perkins, 1994; Réblová and Mostert, 2007; Quijada et al., 2022).

In *Morchella* species, heterothallism, pseudohomothallism and unisexual reproduction modes have been reported not only from different species but also from the same species, such as *M. importuna* (Chai et al., 2017, 2019; Du et al., 2017, 2020; Du and Yang, 2021). To date, only heterothallism has been found in *M. galilaea* (Chai et al., 2019; Du et al., 2020; Du and Yang, 2021), given the variation in spore number in asci and thus the potential for heterokaryotic ascospores formation (especially in those cases of reduced spore number), we presume pseudohomothallism may exist in this species. This awaits verification. As the only species of *Morchella* fruiting in autumn and its wide distribution in Asia, Europe, North American and Africa, the variation in ascospore number in *M. galilaea* might be beneficial in increasing the numbers and diversity of propagules and introduce the potential for the formation of heterokaryotic ascospores aiding in dispersal and long-distance colonization.

**Figure 8:**
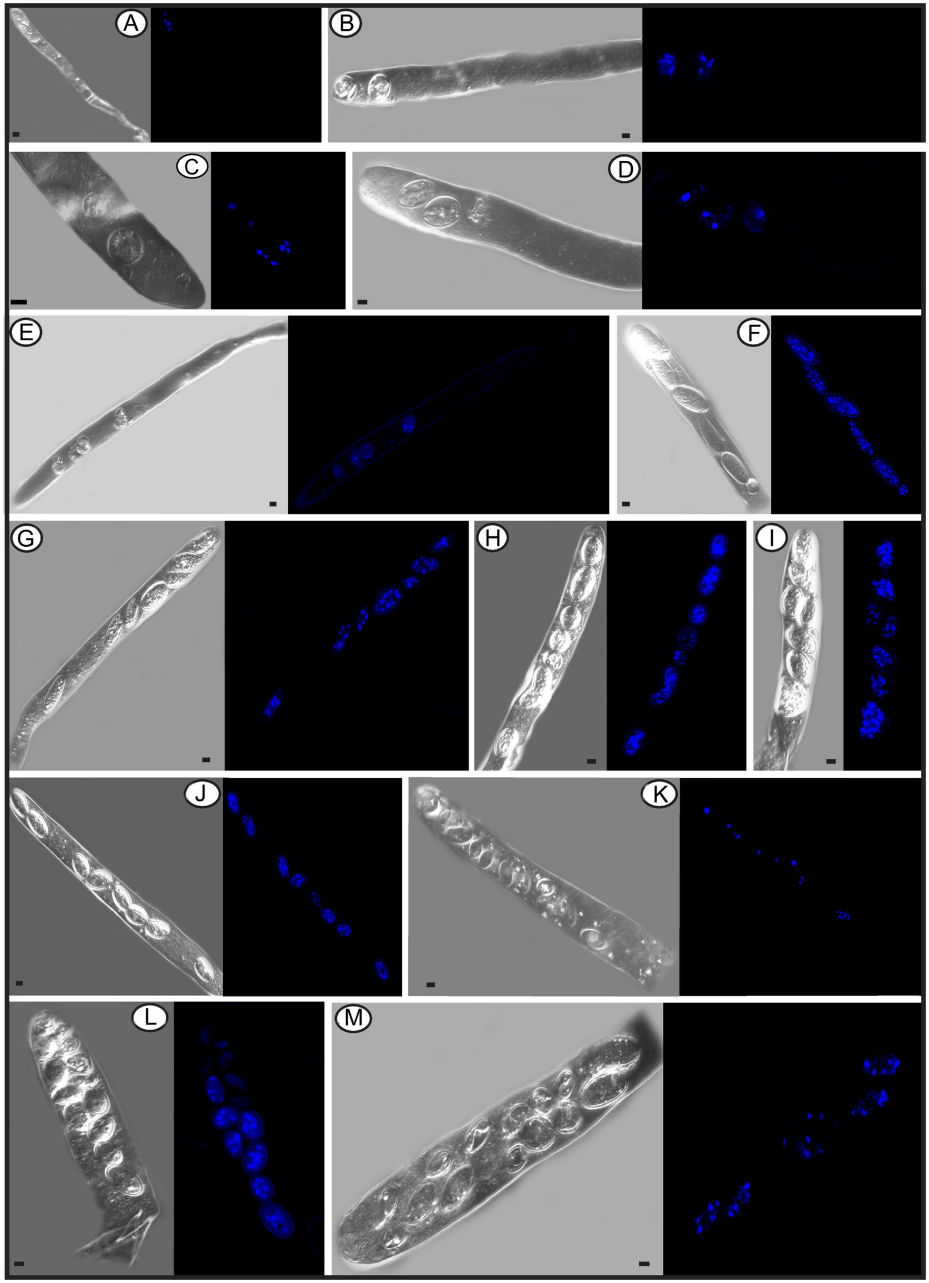
The number of nuclei in one-spored, two-spored, three-spored, four-spored, five-spored, seven-spored, eight-spored, 11-spored, 13-spored and 14-spored asci, and their ascospores of *Morchella galilaea* observed under laser confocal fluorescence microscopy. **(A)**: one-spored ascus with four nuclei (four nuclei in the only spore); **(B)**: two-spored ascus with six nuclei (each spore containing three nuclei); **(C)**: two-spored ascus with eight nuclei (six nuclei in the large spore, one in the small spore; one in the cyptoplasm); **(D)**: three-spored ascus with four nuclei (two nuclei in the large ascospore, two, respectively, in each of two small spores); (E): four-spored ascus with four nuclei (two nuclei in the large ascospore, two in the two other small spores, one aborted spore without nuclei); **(F)**: five-spored ascus with multiple nuclei (each ascospore with four to eight nuclei); **(G–I)**: seven-spored ascus with multiple nuclei (at least four nuclei in each ascospore); **(J)**: eight-spored ascus with multiple nuclei (six to ten nuclei in each ascospore); **(K)**: 11-spored ascus with multiple nuclei (one large ascospore harboring eight nuclei, two spores, respectively, containing three nuclei, one spore with two nuclei, six spores with only one nuclei in each, one aborted spore without nuclei); **(L)**: 13-spored ascus with multiple nuclei (eight ascospores with four to six nuclei, five spores with at least one nuclei in each); **(M)**: 14-spored ascus with multiple nuclei (two ascospores containing six nuclei, two with four nuclei in each, one with two nuclei, one with one nuclei, seven aborted spores without nuclei). Scale bars = 5 μm.

In the Pezizomycetes, the number of nuclei in mature ascospores is considered to have taxonomic value, and varies from 1, 2, 4 to many (Pfister and Healy, 2021). Using laser confocal microscopy we observed the nuclear condition of ascospores in the asci with the typical eight ascospores, and atypical one, two, three, four, five, seven, 11, 13, and 14 spores (Figures 8A–M). For the typical eight-spored asci, usually six to ten nuclei were observed in each ascospore (Figure 8J). In the one-spored ascus (Figure 8A), only four nuclei were observed, indicating the absence of mitosis and/or nuclear degeneration. Although, the possibility also existed of meiosis with cytokinesis defects producing one cell with four nuclei. Two two-spored asci, respectively, possessed six (Figure 8B, three in each spore) and eight nuclei (Figure 8C, six in the large ascospore, one in the small one and one in the cytoplasm). Four nuclei were observed in a three-spored ascus, with two nuclei in a large ascospore and one in each of the small spores (Figure 8D). Four nuclei total in a four-spored ascus were observed, with two nuclei in the large ascospore, two in two other spores, and one spore aborted without a nucleus (Figure 8E). In a five-spored ascus, four to eight nuclei were observed in each spore (Figure 8F). In seven-spored asci (Figures 8G–J), a minimum of four nuclei were observed in each spore, and one moon- and one heart-shaped spore (Figures 8H,I) had almost double the numbers of nuclei (around 16 to 20) compared to typical ascospores. A failure of spore delimitation and cytokinesis defects during mitosis may be responsible for the formation of moon- and heart-shaped spores that otherwise might have divided into pairs of ascospores.

Spore abortion has been suggested to be one of the reasons that fewer than eight spores are found in some asci (Booth, 1959; Rossman, 1983; Glawe and Jacobs, 1988). Spores without nuclei usually fail to reach full maturity or abort after they have been delimited (Dodge, 1928). Based on nuclear observations of two-, three-, and four-spored asci above, larger ascospores usually possess more nuclei than smaller ones (Figures 8C–E). Sherwood (1981) reported that larger spores potentially are multinucleate and may have a heterokaryotic advantage. This may aid in colonization and enhance propagule survival. In pseudohomothallic species, fewer spores and larger ascospores are produced (Raju and Perkins, 1994). The production of multi-nucleate heterokaryotic ascospores gives an advantage in establishment and completion of a species’ life cycle (Uhm and Fujii, 1983; Raju and Perkins, 1994). The pseudohomothallic condition has been demonstrated in *Morchella* species, such as in *M. sextelata*, *M. importuna*, and *Morchella* sp. *Mes*-15 (Liu et al., 2021), but not in *M. galilaea* yet.

For asci with more than eight spores, visualization with laser confocal microcopy requires observations at different angles in order to see all the nuclei. We cannot show a single image in which all nuclei are visible. Therefore, we state how many nuclei were observed in the spores of 11-, 13-, and 14-spored asci, but the corresponding images in Figure 8 may not clearly show the exact number of nuclei. In the 11-spored ascus, one large ascospore harbored eight nuclei, two contained three nuclei, one had two nuclei, six harbored only one nucleus in each and one aborted without nuclei (Figure 8K). For the 13-spored ascus, eight ascospores contained four to six nuclei and the other five spores harbored at least one nucleus in each (Figure 8L). In the 14-spored ascus (Figure 8M), two spores contained six nuclei, two contained four nuclei, one had two nuclei, and one harbored one nucleus, but the other seven aborted spores lacked nuclei even at different observational angles.

In contrast to asci with more or fewer than eight spores, eight-spored asci in *M. galilaea* have more nuclei per spore, usually six to ten and even more. Ascospores with no nuclei (aborted) or fewer than six nuclei were commonly found. In ascomycetes, the fusion nucleus in the ascus regularly divides in succession by meiosis and post-meiotic mitosis to produce eight nuclei before spore delimitation for typical eight-spored asci (Dodge, 1928; Pöggeler et al., 2006); for asci with more than eight spores, the eight post-mitotic nuclei undergo further successive mitotic divisions so that 16, 32, or more nuclei are formed before spore delimitation (Dodge, 1928). Spore fragmentation to form part spores and budding of ascospores within asci may be also responsible for more than eight spores being found in asci in some fungi (Read and Beckett, 1996). When there are fewer than eight ascospores per ascus, nuclei degeneration followed by spore abortion, an absence of mitotic divisions, or multiple nuclei encompassed by a single wall to form a giant, multinucleate spore may be the reasons (Esser and Stahl, 1976; Leonard, 1988; Read and Beckett, 1996), as we observed in one-, two–, three– and four-spored asci in *M. galilaea*. Among all the different asci observed in *M. galilaea*, a minimum of four nuclei in developing asci was observed, except the diploid state with a single nucleus present, and nuclear number in spores can vary from zero to around 20. The four nucleate states observed in some asci suggest that meiosis occurs, but whether post-meiotic mitosis follows needs to be verified. Further studies of meiotic and mitotic events correlated with nuclear status in asci and spore delimitation are needed in the future.

#### 3.3.3. Relationship among ascospore size, ascospore number, and ascus size in *M*orchella *galilaea*

A negative correlation exists between ascospore size and the number of ascospores per ascus in most ascomycetes, i.e., production of fewer than eight ascospores per ascus is correlated with a maximum spore size whereas having more than eight ascospores is correlated with a reduced spore size (Sherwood, 1981). Ascus size was also shown to correlate with ascospore size in the Nectriaceae (Hirooka et al., 2012). To probe the relationships among ascospore number, ascospore dimension and ascus size, we measured the length and width of ascospores in one- to 16-spored asci observed from *M. galilaea*, as well as the length and width of the asci (Supplementary Table S2).

**Figure 9:**
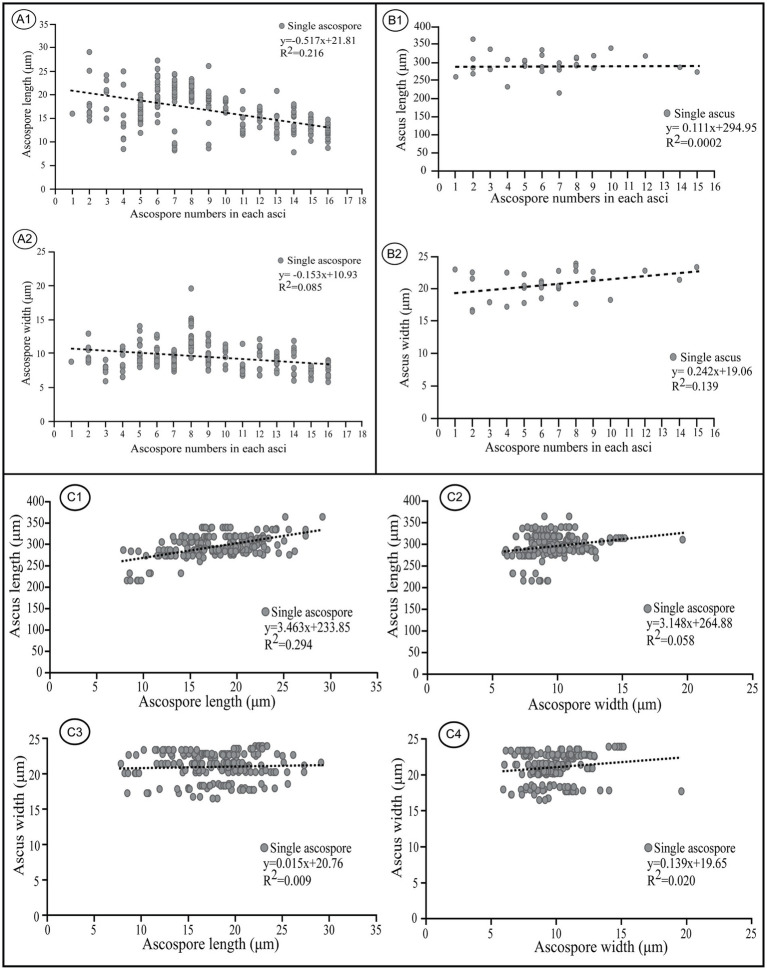
Correlations between ascospore size, number, and the corresponding ascus size of *Morchella galilaea*. **(A)**: correlation between ascospore length, width, and spore number in the ascus; **(B)**: correlation between ascus length, width, and spore number in the ascus; **(C)**: correlation between ascospore length, width and the ascus size.

Negative linear correlations were apparent in a scatter diagram for length and width of ascospores within asci and ascospore number (r = −0.517 for ascospore length, r = −0.153 for ascospore width) (Figure 9A); this agrees with previous observations (Sherwood, 1981). Coefficient of determination (R^2^) values indicated that 21.6% of the variability in ascospore length may be explained by ascospore number which also was responsible for 8.5% of the observed variability in ascospore width (Figure 9A). In contrast, positive linear correlations were indicated for the length and width of asci and ascospore number within the corresponding asci (r = 0.111 for asci length, r = 0.242 for asci width) in which the variable ascospore number influenced ascus width more than length (Figure 9B). Coefficient of determination (R^2^) values indicated that only 0.02% of the variability in ascus length was explained by ascospore number, which was responsible for 13.9% of the observed variability in ascus width (Figure 9B).

Apparent positive linear correlations were shown for ascus length and ascospore size (r = 3.463 for ascospore length, r = 3.148 for ascospore width, Figure 9, C1 and C2). Coefficient of determination (R^2^) values indicated that 29.4% of the variability in ascus length may be explained by ascospore length, and 5.8% may be explained by ascospore width. Weak positive linear correlations were shown for ascus width and ascospore size (r = 0.015 for ascospore length, r = 0.139 for ascospore width, Figure 9, C3 and C4). Coefficient of determination (R^2^) values indicated that 0.09% of the variability in ascus length was due to ascospore length, and 2% was related to ascospore width.

In *M. galilaea*, ascus size is influenced more by ascospore length than ascospore width, which is probably in accordance with the similar shapes of asci (cylindric) and ascospores (elliptical). The production of fewer than eight spores in asci increases maximum potential spore size. Large, compartmentalized spores have clear advantages in a harsh environment and also increase impaction efficiency and the amount of mycelium which could be produced (Sherwood, 1981). Large spores in fewer than eight-spored asci have more chance for survival, but it may impede active discharged from asci. Their generation size also decreases. The large number of spores produced in polysporous asci decrease spore size, but increase their generation size and also survival possibility of offspring even if some spores fail to survive. Both conditions have some adaptive values, but the production of eight-spored asci probably has the optimal value suitable for ascus discharge mechanism and the ascospore size needed to harbor nuclei and other organelles to survive.

#### 3.3.4. Ascospore germination and conidial formation in *M*orchella *galilaea*

**Figure 10:**
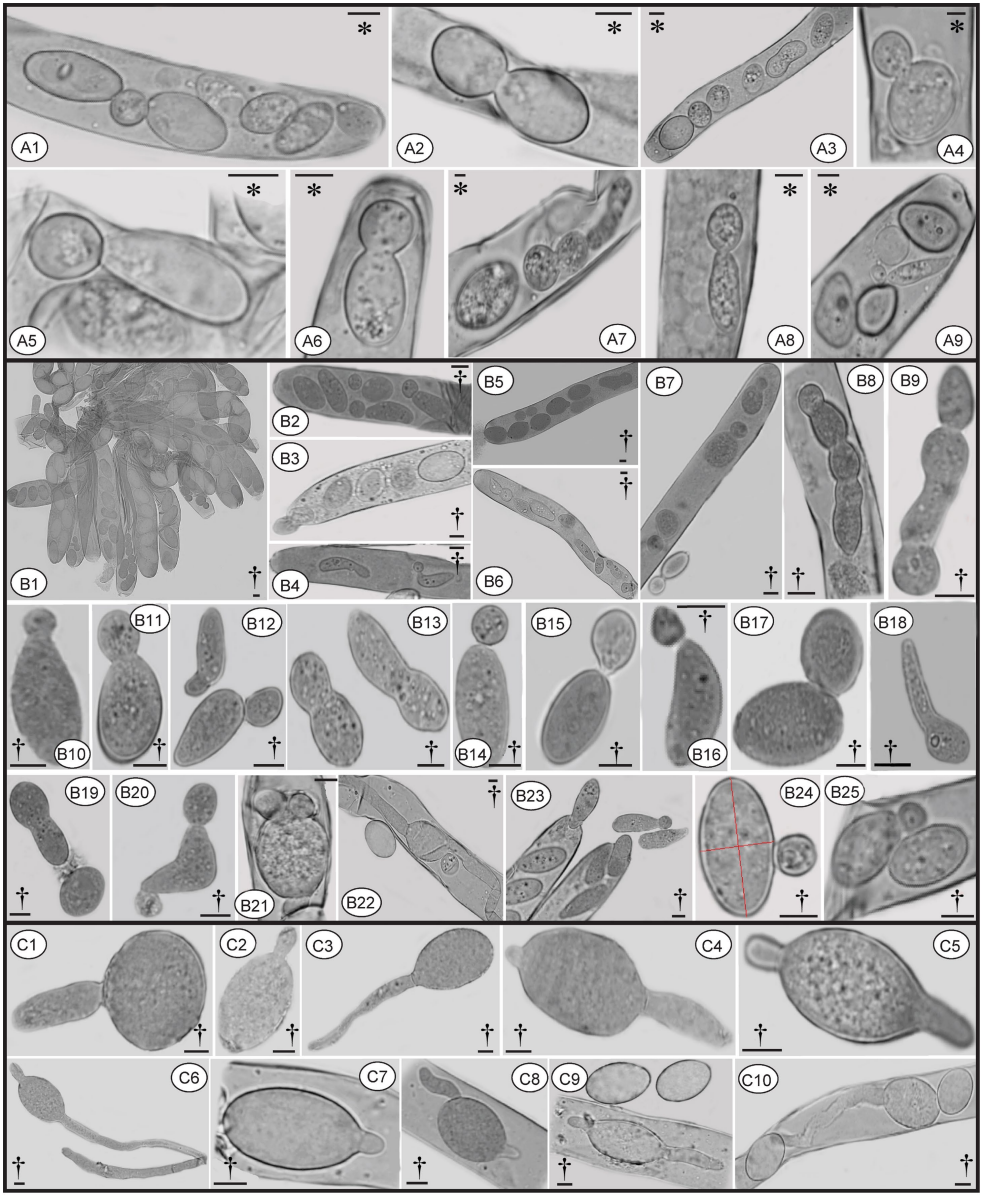
Conidia formation from ascospores from fresh **(A)** and dried specimens **(B)** of *Morchella galilaea*; ascospore germination by long germ tubes (and via conidia) **(C)**. Symbols in the figure panels (* = fresh specimen, † = dried specimen) and terminology used following Baral (1999). **(A)**: conidia produced by the fresh specimen: **(A1-A7)**: conidia formation from ascospores in living asci; **(A8,A9)**: mature conidia detached from ascospores. **(B)**: conidia produced by dried specimens as follows: **(B1–B7)**: conidia formation from ascospores in asci; **(B8)**: conidia in chains in the ascus; **(B9)**: conidia in chains outside the ascus; **(B10–B13)**: conidia from ascospores outside asci; **(B14–B17)**: conidia detached from the ascospore outside asci; **(B18)**: potential conidium produced outside asci; **(B19)**: conidia detached from the ascospore; **(B20)**: two conidia produced from both ends of the ascospore; **(B21)**: three conidia produced from both spore ends of the ascospore in asci; **(B22)**: one conidium and two germ tubes produced by one ascospore in the ascus; **(B23)**: conidia; **(B24)**: conidium produced laterally from the ascospore. **(C)**: ascospore germination from dry samples: **(C1–C6)**: ascospore germination with one or two germ tubes outside asci; **(C7–C9)**: ascospore germination in asci with one or two germ tubes; **(C10)**: both one germ tube and one conidium produced from one ascospore in asci. Scale bars = 5 μm.

Two types of ascospore germination were observed in *M. galilaea.* Ascospores germinate via a single, long germ tube or by the direct production of conidia from ascospores, or by both simultaneously. Ascospores may germinate within and outside asci. Production of conidia directly from ascospores without the typical intervening mycelial phase was investigated here from one freshly collected and four dried specimens, results are shown in Table 1. Among the five specimens (Table 1), conidial formation was observed not only in ascospores within living asci from the freshly collected specimen (Figure 10A), but also from ascospores within dead asci or after discharge from the three dried specimens (Figure 10B), however, no conidia were found arising directly from ascospores from the specimen from Kenya.

Ascospores in *M. galilaea* can germinate terminally at one or both spore poles, or laterally at multi-sites, and by formation of hypha and/or conidia singly or in sequence. The types of ascospore germination are shown in Figure 10. Mature ascospores may be ejected from asci with conidia attached (Figures 10B3 and 11C4). Once conidia mature, they may gradually detach from ascospores within (Figure 10A2,A9,B2,B4) or outside of the asci (Figure 10B14–B16).

**Figure 11:**
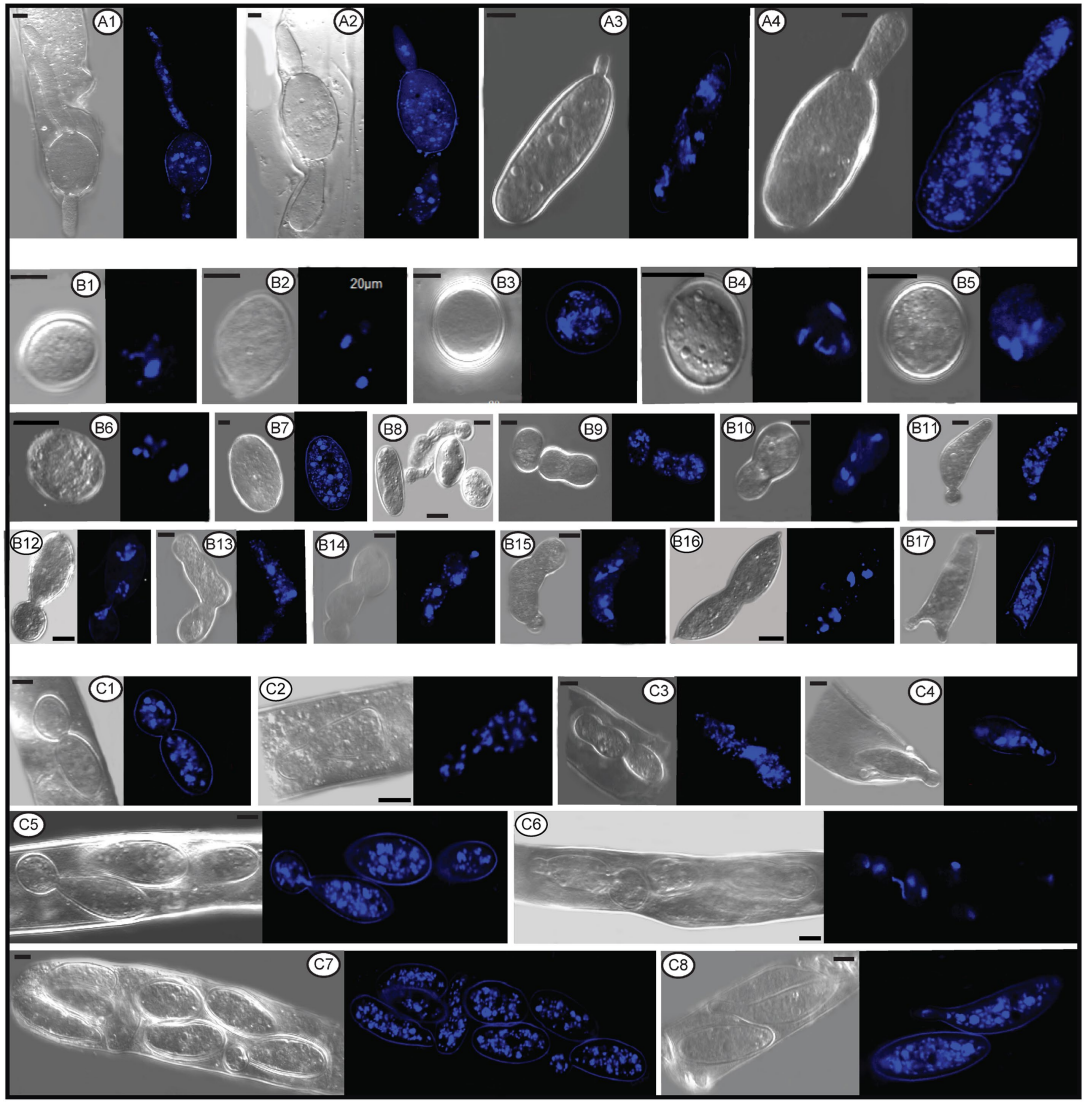
The number and status of nuclei in conidia, germ tube, and ascospores within or outside asci of *Morchella galilaea* by laser confocal fluorescence microscopy. **(A1–A4)**: nuclei in germ tubes and ascospores; **(B1–B6)**: nuclei in potential detached conidia; **(B7)**: nuclei in the normal ascospore in contrast with conidia; **(B8–B17)**: nuclei in conidia single or sequentially produced by budding from ascospores outside acsi, with nuclei migration through bud necks from ascospores to conidia (and further to sequential conidia) noted; **(C1–C8)**: nuclei in conidia budding from ascospores in asci, with nuclei migration through bud necks from ascospores to conidia noted. Scale bars = 5 μm.

**Figure 12:**
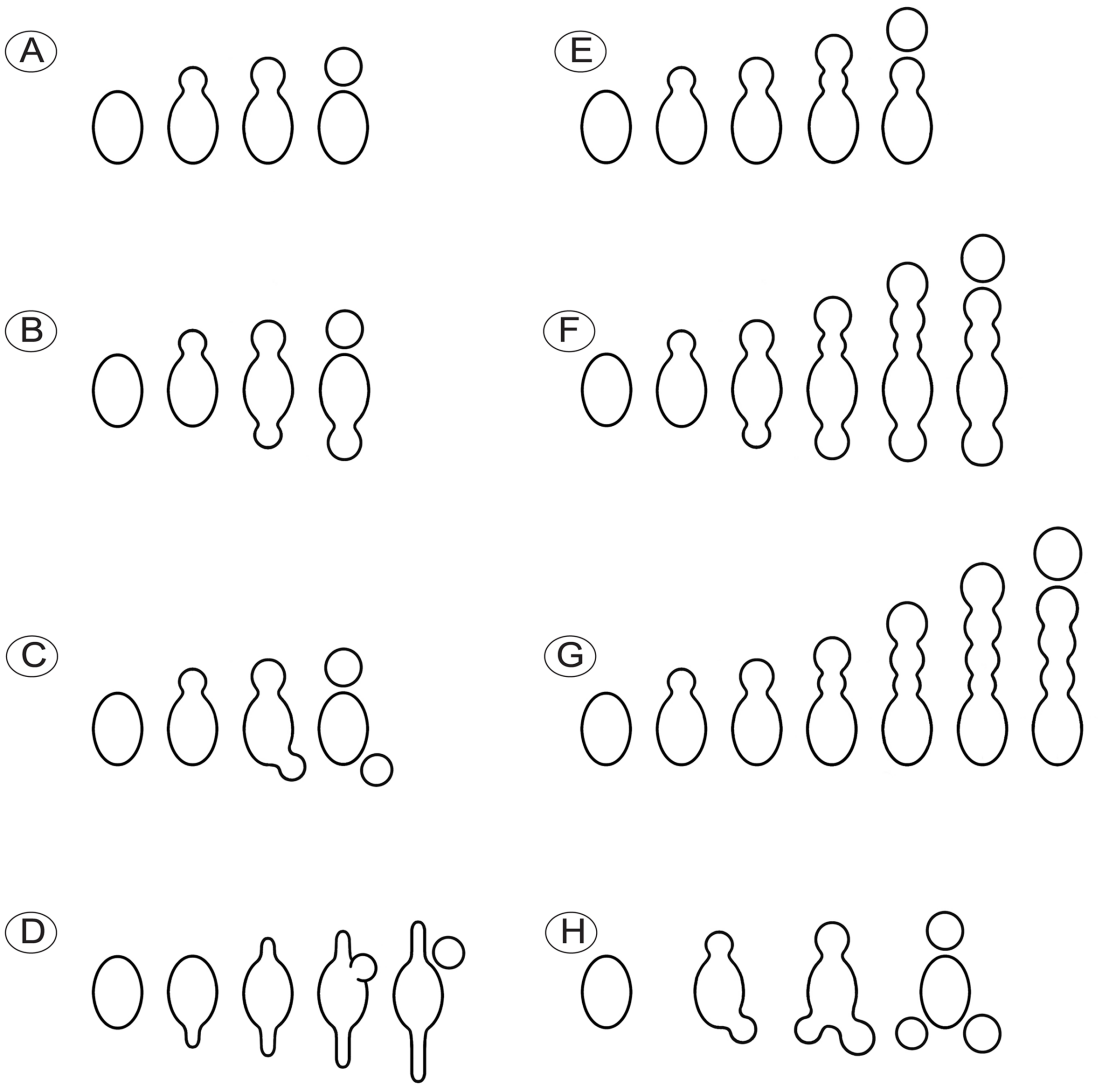
Schematic diagram illustrating the ontogeny of conidia singly or sequentially produced by budding from ascospores in *Morchella galilaea*. **(A)**: conidium produced singly at one spore end; **(B,C)**: two conidia produced singly at both spore ends; **(D)**: conidium singly with both germ tubes produced by one ascospore; **(E–G)**: different types of sequential conidia produced from one or both ends of ascospores; **(H)**: three conidia produced at both spore ends of one ascospore.

Conidia were produced from about 0.01% of ascospores based on observations from three dried specimens (1/1016, 1/1029, 1/1015). The hyaline conidia in *M. galilaea* were globose or occasionally elliptical, ranging in diameter from approximately 2–10 μm (Figure 10). The ontogeny of conidial emergence from ascospores to the detachment of single and sequential conidia is illustrated schematically based on our microscopy observations (Figure 12). More morphological types of conidia formation are presented in Figure 4B2, C1-C7.

The nuclear status of ascospores at germination either by long germ-tubes (Figure 11A) or by production of conidia within or outside asci (Figures 11B,C) was further investigated. Migration of nuclei from ascospores through the bud necks into conidia was observed (Figure 11C2,C4–C6), but whether further nuclear mitotic divisions occur in conidia before detachment is unverified. One to six nuclei were observed from detached (Figure 11B1–B6) and attached conidia (Figure 11B8–C8), which was in sharp contrast to 14 nuclei noted in the ascospore (Figure 11B7). Nuclear migration through the bud neck from the primary conidium into successive conidia also was noted (Figure 11B14,B15).

In species of the genus *Morchella*, conidia are produced on upright, aerial conidiophores after a period of vegetative growth during their hyphal development (this state was previously identified as *Costantinella*). These conidia are spherical and 2.5–8 μm in diameter, and harbor one to three nuclei (Carris et al., 2015; Yuan et al., 2021; Pfister et al., 2022). The size, nuclear status and formation of conidia produced directly from ascospores are similar to conidia that are produced from mycelium. The similar morphology of conidia produced directly from spores and from aerial hyphae was also described in *Nectria inaurata* and was found in other fungi (Brefeld, 1891). These common features suggest that development of both modes of conidial formation differ primarily in the timing of conidial initiation in the life cycle, and thus further indicate the two kinds of conidia may have similar functions, such as serving as colonial propagules or as spermatia.

Among the five samples of *M. galilaea* used in this study, four collections from China exhibited conidia produced from ascospores, whereas the sample from Kenya contained mature ascospores, but their ascospores did not produce conidia. Phylogenetic analyses supported all the five samples as *M. galilaea* with high bootstrap (100%) (Figure 2). The current results suggested that conidial production from ascospores seems not to be a useful taxonomic feature in *M. galilaea*, but new markers and additional samples in further research on this species will likely provide more definitive information. That the sample from Kenya lacks conidia produced from ascospores may suggest emerging morphological difference that are due to geographic isolation (Figure 2) but caution is warranted since only a single sample from Kenya was used in this study.

Conidia produced directly from ascospores have rarely been reported in the Pezizomycetes, but have been noted in *P. subisabellina* (Van Vooren, 2020) and members of the Sarcoscyphaceae (Paden, 1975). *Morchella galilaea* is the first species reported in the Morchellaceae to produce conidia directly from ascospores. Given that seemingly no special conditions are required during conidial production directly from ascospores, it is likely that this phenomenon is widely distributed but overlooked in fungi. Our findings call for more research into investigating conidial production directly from meiospores.

Production of ascoconidia and conidia directly from ascospores often correlate with ecological niche (Ingold, 1975; Campbell et al., 2006; Shenoy et al., 2006). Such development has been suggested to be associated with drought-tolerance (Sherwood, 1981); colonization of new nutrient sources for coprophilous fungi (Quijada et al., 2022); infection of new hosts in harsh environments (Juzwik and Hinds, 1984; Hanlin, 1994) and performing the role of spermatia (Hanlin, 1994). The broad distribution of *M. galilaea* throughout Asia, Europe, North America, and Africa, and the autumn fruiting of the species (Du et al., 2015; Taşkin et al., 2015), suggests strong selective pressures from environmental and habitat factors that may favor rapid dispersal and colonization by means of diverse and numerous propagules. These propagules may serve as spermatia and be involved in sexual reproduction during the special fruiting timeline of this fungus. It is important to note that ascomata of species of *Morchella* are primarily produced in the spring, but their anamorphic spore mats are mainly produced in the fall. These spore mats were not only reported from the Northern Hemisphere (Carris et al., 2015), but also in the Southern Hemisphere (Pfister et al., 2022). So far, field-collected anamorphs have not been reported for *M. galilaea*. The function of conidia produced from ascospores in *M. galilaea* still requires further investigation.

## 4. Conclusion

Despite the broad geographical distribution of species in the genus *Morchella*, the life history of the species are still far from well known. The information presented here on direct conidial production from ascospores and variability in ascospore shape and number in *M. galilaea*, suggests that there is aberration during spore formation and/or that there is a selective advantage in these features. Further research is needed to elucidate these factors. That this species produces ascomata in the fall, unlike other species, and that it uniquely produces conidia directly from ascospores suggests that further research may cast light on life histories of the species of *Morchella* in general. Our findings call special attention to the occurrence of conidia produced directly from ascospores among clades within Pezizomycetes, and also expand the current known morphological and lifestyle diversity in this class.

## Data availability statement

The datasets presented in this study can be found in online repositories. The names of the repository/repositories and accession number(s) can be found in the article/Supplementary material.

## Author contributions

X-HD: Conceptualization, Data curation, Formal analysis, Funding acquisition, Investigation, Methodology, Project administration, Resources, Software, Supervision, Validation, Visualization, Writing – original draft, Writing – review & editing. S-YW: Methodology, Writing – review & editing. MR: Writing – review & editing. Y-JG: Resources, Writing – review & editing. J-YW: Resources, Writing – review & editing. DP: Supervision, Writing – review & editing, Conceptualization. HJ: Supervision, Writing – review & editing, Conceptualization.

## References

[ref1] Adams T. H. Wieser J. K. Yu J. H. (1998). Asexual sporulation in *aspergillus nidulans*. Microbiol. Mol. Biol. Rev. 62, 35–54. doi: 10.1128/MMBR.62.1.35-54.1998, PMID: 9529886 PMC98905

[ref2] Baral H. O. (1999). A monograph of *Helicogonium* (=*Myriogonium*, *Leotiales*), a group of non-ascocarpous intrahymenial mycoparasites. Nova Hedwig. 69, 1–71. doi: 10.1127/nova.hedwigia/69/1999/1

[ref3] Bonito G. Trappe J. M. Rawlinson P. Vilgalys R. (2010). Improved resolution of major clades within *tuber* and taxonomy of species within the *tuber gibbosum* complex. Mycologia 102, 1042–1057. doi: 10.3852/09-213 20943504

[ref4] Booth C. (1959). Studies of Pyrenomycetes IV. *Nectria* (part I). Mycol. Papers. 73, 1–115.

[ref5] Braus G. H. Krappmann S. Eckert S. E. (2002). Sexual development in ascomycetes: fruit body formation of *aspergillus nidulans*. Mycol. Series. 15, 215–244. doi: 10.1201/9780203910719.ch9

[ref6] Brefeld O. (1891). Untersuchungen aus dem gesammtgebiete der Mykologie. Ascomyceten. II. Muenster. 9, 164–170.

[ref7] Campbell J. Shearer C. Marvanová L. (2006). Evolutionary relationships among aquatic anamorphs and teleomorphs: *Lemonniera*, *Margaritispora*, and *Goniopila*. Mycol. Res. 110, 1025–1033. doi: 10.1016/j.mycres.2006.04.012, PMID: 16930975

[ref8] Carris L. M. Peever T. L. McCotter S. W. (2015). Mitospore stages of *Disciotis*, *Gyromitra* and *Morchella* in the inland Pacifc Northwest USA. Mycologia 107, 729–744. doi: 10.3852/14-207, PMID: 25911699

[ref9] Chai H. Chen L. Chen W. Zhao Q. Zhang X. Su K.. (2017). Characterization of mating-type idiomorphs suggests that *Morchella importuna*, *Mel*-20 and *M. Sextelata* are heterothallic. Mycol. Progress. 16, 743–752. doi: 10.1007/s11557-017-1309-x

[ref10] Chai H. Chen W. Zhang X. Su K. Zhao Y. (2019). Structural variation and phylogenetic analysis of the mating-type locus in the genus *Morchella*. Mycologia 111, 551–562. doi: 10.1080/00275514.2019.1628553 31251705

[ref11] Chaudhary V. B. Aguilar-Trigueros C. A. Mansour I. Rillig M. C. (2022). Fungal dispersal across spatial scales. Annu. Rev. Ecol. Evol. Syst. 53, 69–85. doi: 10.1146/annurev-ecolsys-012622-021604

[ref12] Clowez P. Alvarado P. Becerra M. Bilbao T. Moreau P. A. (2014). *Morchella fluvialis* sp. nov. (Ascomycota, Pezizales): a new but widespread morel in Spain. Boletne. De. La. Sociedad. Micollad. M. de. Madrid. 38, 253–262.

[ref13] Clowez P. Bellanger J. M. Romero O. L. Moreau P. A. (2015). *Morchella palazonii* sp. nov. (Ascomycota, Pezizales): une nouvelle morille méditerranéenne. Clé des *Morchella* sect. Morchella en Europe. Doc. Mycol. 36, 71–84.

[ref14] Davidow L. S. Goetsch L. (1978). Spindle pole body regulation of spore wall formation in yeast. J. Cell Biol. 79:1114.

[ref15] Dodge B. O. (1928). Spore formation in asci with fewer than eight spores. Mycologia 20, 18–21. doi: 10.1080/00275514.1928.12016891

[ref16] Du X. H. Wu D. M. He G. Q. Wei W. Xu N. Li T. L. (2019). Six new species and two new records of *Morchella* in China using phylogenetic and morphological analyses. Mycologia 111, 857–870. doi: 10.1080/00275514.2019.1640012, PMID: 31414967

[ref17] Du X. H. Wu D. Kang H. Wang H. Xu N. Li T.. (2020). Heterothallism and potential hybridization events inferred for twenty-two yellow morel species. IMA Fungus. 11:4. doi: 10.1186/s43008-020-0027-1, PMID: 32617256 PMC7325075

[ref18] Du X. H. Yang Z. L. (2021). Mating systems in true morels (*Morchella*). Microbiol. Mol. Biol. Rev. 85:e00220. doi: 10.1128/MMBR.00220-20 34319143 PMC8483713

[ref19] Du X. H. Zhao Q. O'Donnell K. Rooney A. P. Yang Z. L. (2012). Multigene molecular phylogenetics reveals true morels (*Morchella*) are especially species-rich in China. Fungal Genet. Biol. 49, 455–469. doi: 10.1016/j.fgb.2012.03.006, PMID: 22503770

[ref20] Du X. H. Zhao Q. Xia E. H. Gao L. Z. Richard F. Yang Z. L. (2017). Mixed-reproductive strategies, competitive mating-type distribution and life cycle of fourteen black morel species. Sci. Rep. 7:1493. doi: 10.1038/s41598-017-01682-8, PMID: 28473711 PMC5431422

[ref21] Du X. H. Zhao Q. Yang Z. L. (2015). A review on research advances, issues, and perspectives of morels. Mycology 6, 78–85. doi: 10.1080/21501203.2015.1016561, PMID: 30151316 PMC6106076

[ref22] Ertz D. Diederich P. (2004). Revision of *Trimmatothele* (Verrucariaceae), and description of *Oevstedalia* for *Trimmatothelopsis Antarctica*, a new lichen genus with true ascoconidia. Mycol. Progress. 3, 229–236. doi: 10.1007/s11557-006-0093-9

[ref23] Esser K. Stahl U. (1976). Cytological and genetic studies of the life cycle of *Saccharomycopsis lipolytica*. Mol. Genet. Genomics 146, 101–106. doi: 10.1007/BF00267989, PMID: 785204

[ref24] Frisch A. Klaus K. (2006). The lichen genus *Topeliopsis*, additions and corrections. Lichenologist 38, 37–45. doi: 10.1017/S0024282905005530

[ref25] Gardes M. Bruns T. D. (1993). ITS primers with enhanced specificity for basidiomycetes-application to the identification of mycorrhizae and rusts. Mol. Ecol. 2, 113–118. doi: 10.1111/j.1365-294X.1993.tb00005.x, PMID: 8180733

[ref26] Glawe D. A. Jacobs K. A. (1988). Evolutionary aspects of ascospore and conidium ontogeny in *Scoleconectria cucurbitula*. Mycologia 80, 636–645. doi: 10.1080/00275514.1988.12025594

[ref27] Goh Y. K. Daida P. Vujanovic V. (2009). Effects of abiotic factors and biocontrol agents on chlamydospore formation in *fusarium graminearum* and *fusarium sporotrichioides*. Biocontrol Sci. Tech. 19, 151–167. doi: 10.1080/09583150802627033

[ref28] Hall T. A. (1999). BioEdit: a user-friendly biological sequence alignment editor and analysis program for windows 95/98/NT. Nucleic Acids Symp. Ser. 41, 95–98. doi: 10.12691/ajmr-3-2-1

[ref29] Hanlin R. T. (1994). Microcycle conidiation- a review. Mycoscience 35, 113–123. doi: 10.1007/BF02268539

[ref30] Hawksworth D. L. (1987). “The evolution and adaptation of sexual reproductive structures in the Ascomycotina” in Evolutionary biology of the Fungi. eds. Rayner A. D. M. Brasiel C. M. Moore D. (Cambridge: Cambridge Univ. Press), 179–189.

[ref31] Hawksworth D. L. Kirk P. M. Sutton B. C. Pegler D. N. (1995). Ainsworth & Bisby’s dictionary of the Fungi. 8th Wallingford, UK: CAB International. 616.

[ref32] Healy R. Pfister D. H. Rossman A. Y. Marvanová L. Hansen K. (2016). Competing sexual-asexual generic names of Pezizomycetes and recommendations for use. IMA fungus. 7, 285–288. doi: 10.5598/imafungus.2016.07.02.08, PMID: 27990335 PMC5159599

[ref33] Hirooka Y. Rossman A. Y. Samuels G. J. Lechat C. Chaverri P. (2012). A monograph of *Allantonectria*, *Nectria*, and *Pleonectria* (*Nectriaceae*, *Hypocreales*, *Ascomycota*) and their pycnidial, sporodochial, and synnematous anamorphs. Stud. Mycol. 71, 1–210. doi: 10.3114/sim0001, PMID: 22685364 PMC3310236

[ref34] Huang S. K. Hyde K. D. Mapook A. Maharachchikumbura S. S. N. Bhat J. D. McKenzie E. H. C.. (2021). Taxonomic studies of some often overlooked Diaporthomycetidae and Sordariomycetidae. Fungal Divers. 111, 443–572. doi: 10.1007/s13225-021-00488-4

[ref35] Ingold C. T. (1975). An illustrated guide to aquatic Hyphomycetes. Sci. Publ. Freshw. Biol. Assoc, 30, 96.

[ref36] Juzwik J. Hinds T. E. (1984). Ascospore germination, mycelial growth, and microconidial anamorphs of *Encoelia pruinosa* in culture. Canad. J. Bot. 62, 1916–1919. doi: 10.1139/b84-261

[ref37] Karakehian J. M. Quijada L. Pfister D. H. Tocci G. E. Miller A. N. (2021). Methods for observing, culturing, and studying living ascospores. Asian J. Mycol. 4, 1–18. doi: 10.5943/ajom/4/2/1

[ref38] Katoh K. Misawa K. Kuma K. Miyata T. (2002). MAFFT: a novel method for rapid multiple sequence alignment based on fast Fourier transform. Nucleic Acids Res. 30, 3059–3066. doi: 10.1093/nar/gkf436, PMID: 12136088 PMC135756

[ref39] Kauserud H. Schumacher T. (2001). Outcrossing or inbreeding: DNA markers provide evidence for type of reproductive mode in *Phellinus nigrolimitatus* (Basidiomycota). Mycol. Res. 105, 676–683. doi: 10.1017/S0953756201004191

[ref40] Kušan I. Matočec N. Jadan M. Tkalčec Z. Armin Mešić A. (2018). An overview of the genus *Coprotus* (Pezizales, Ascomycota) with notes on the type species and description of *C*. epithecioides sp. nov. MycoKeys. 29, 15–47. doi: 10.3897/mycokeys.29.22978, PMID: 29559824 PMC5804121

[ref41] Lechat C. Gardiennet A. Fournier J. (2018). *Thyronectria abieticola* (Hypocreales), a new species from France on *Abies alba*. Ascomycete org. 10, 55–61. doi: 10.25664/ART-0228

[ref42] Leonard K. J. (1988). *Setosphaeria turcica*, cause of northern corn leaf blight, and other *Setosphaeria* spp. Adv Plant Pathol. 6, 241–248. doi: 10.1016/B978-0-12-033706-4.50020-7

[ref43] Liu W. Cai Y. He P. Zhang Y. Bian Y. (2016). Morphological and structural analysis of mitospores of *Morchella importuna*. J. Fungal Res. 14, 157–161. doi: 10.13341/j.jfr.2014.1099

[ref44] Liu P. Ma Y. Zhao Y. Chai H. Li Y. (2021). Sexual and vegetative compatibility of single ascospores isolations in the genus *Morchella*. Acta Edulis Fungi. 28, 40–47. doi: 10.16488/j.cnki.1005-9873.2021.01.006

[ref45] Loizides M. Bellanger J. M. Clowez P. Richard R. Moreau P. A. (2016). Combined phylogenetic and morphological studies of true morels (Pezizales, Ascomycota) in Cyprus reveal significant diversity, including *Morchella arbutiphila* and *M. disparilis* spp. nov. Mycol. Prog. 15, 1–28. doi: 10.1007/s11557-016-1180-1

[ref46] Molliard M. (1904). Mycelium et forme conidienne de la morille. C. R. Hebd. Seances Acad. Sci. 138, 516–517.

[ref47] Neiman A. M. (2005). Ascospore formation in the yeast *Saccharomyces cerevisiae*. Microbiol. Mol. Biol. Rev. 69, 565–584. doi: 10.1128/MMBR.69.4.565-584.2005, PMID: 16339736 PMC1306807

[ref48] Nieuwenhuis B. P. James T. Y. (2016). The frequency of sex in fungi. Philos. T. R. Soc. B. 371:20150540. doi: 10.1098/rstb.2015.0540, PMID: 27619703 PMC5031624

[ref49] O’Donnell K. Rooney A. P. Mills G. L. Kuo M. Weber N. S. Rehner S. A. (2011). Phylogeny and historical biogeography of true morels (*Morchella*) reveals an early cretaceous origin and high continental endemism and provincialism in the Holarctic. Fungal Genet. Biol. 48, 252–265. doi: 10.1016/j.fgb.2010.09.006, PMID: 20888422

[ref50] Okuda S. (1961). Sporulation of the yeast, *Hansenula saturnus* ii. Influence of carbon-nitrogen ratio of culture medium. Plant Cell Physiol. 2, 371–381. doi: 10.1093/oxfordjournals.pcp.a077693

[ref51] Paden J. W. (1975). Ascospore germination, growth in culture, and imperfect spore formation in *Cookeina sulcipes* and *Phillipsia crispata*. Canad. J. Bot. 53, 56–61. doi: 10.1139/b75-008

[ref52] Pfister D. H. Healy R. (2021). Pezizomycetes. Encyclopedia mycol. 1, 295–309. doi: 10.1016/B978-0-12-819990-9.00054-8

[ref53] Pfister D. H. Healy R. LoBuglio K. F. Furci G. Mitchell J. Smith M. E. (2022). South American morels in the Elata group: mitosporic states, distributions, and commentary. Mycol. Prog. 21:97. doi: 10.1007/s11557-022-01846-5

[ref54] Pöggeler S. Nowrousian M. Kück U. (2006). In Growth, differentiation and sexuality. Berlin, Heidelberg: Springer Berlin Heidelberg. 325–355.

[ref55] Quijada L. (2015). Estudio de los órdenes Helotiales s.l. y Orbiliales (Ascomycota, Fungi) en la Isla de Tenerife. Ph.D. dissertation, Departamento de Botánica, Ecología y Fisiología Vegetal, Universidad de La Laguna, España.

[ref56] Quijada L. Matočec N. Kušan I. Tanney J. B. Johnston P. R. Mešić A.. (2022). Apothecial ancestry, evolution, and re-evolution in Thelebolales (Leotiomycetes, Fungi). Biol. 11:583. doi: 10.3390/biology11040583 PMC9026407 35453781

[ref57] Quijada L. Mitchell J. K. Popov E. Pfister D. H. (2019). The Asian-Melanesian bambusicolous genus *Myriodiscus* is related to the genus *Tympanis*, the north American-European tree pathogen. For. Pathol. 49:e12532. doi: 10.1111/efp.12532

[ref58] Raju N. B. Burk A. G. (2004). Abnormal ascospore morphology in the bud mutant of *Neurospora tetrasperma*. Fungal Genet. Biol. 41, 582–589. doi: 10.1016/j.fgb.2004.01.007, PMID: 15121081

[ref59] Raju N. B. Perkins D. D. (1994). Diverse programs of ascus development in pseudohomothallic species of *Neurospora*, *Gelasinospora*, and *Podospora*. Dev. Genet. 15, 104–118. doi: 10.1002/dvg.1020150111, PMID: 8187347

[ref60] Ramaley A. W. (1997). *Barrina*, a new genus with polysporous asci. Mycologia 89, 962–966. doi: 10.1080/00275514.1997.12026868

[ref61] Read N. D. Beckett A. (1996). Ascus and ascospore morphogenesis. Mycol. Res. 100, 1281–1314. doi: 10.1016/S0953-7562(96)80057-8

[ref62] Réblová M. Jaklitsch W. M. Réblová K. Štěpánek V. (2015). Phylogenetic reconstruction of the Calosphaeriales and Togniniales using five genes and predicted RNA secondary structures of ITS, and *Flabellascus tenuirostris* gen. Et sp. nov. PLoS One 10:e0144616. doi: 10.1371/journal.pone.0144616, PMID: 26699541 PMC4689446

[ref63] Réblová M. Mostert L. (2007). *Romellia* is congeneric with *Togninia*, and description of *Conidiotheca* gen. Nov. for one species of this genus with polysporous asci. Mycol. Res. 111, 299–307. doi: 10.1016/j.mycres.2006.12.002, PMID: 17350241

[ref64] Réblová M. Štěpánek V. (2018). Introducing the Rhamphoriaceae, fam. Nov. (Sordariomycetes), two new genera, and new life histories for taxa with *Phaeoisaria*- and *Idriella*-like anamorphs. Mycologia 110, 750–770. doi: 10.1080/00275514.2018.1475164, PMID: 30125239

[ref65] Rehner S. A. Buckley E. (2005). A *Beauveria* phylogeny inferred from nuclear ITS and EF1-α sequences: evidence for cryptic diversification and links to *Cordyceps teleomorphs*. Mycologia 97, 84–98. doi: 10.3852/mycologia.97.1.84, PMID: 16389960

[ref66] Ronquist F. Teslenko M. van der Mark P. Ayres D. L. Darling A. Hohna S.. (2012). MrBayes 3.2: efficient bayesian phylogenetic inference and model choice across a large model space. Syst. Biol. 61, 539–542. doi: 10.1093/sysbio/sys029, PMID: 22357727 PMC3329765

[ref67] Roper M. Petter R. E. Brenner M. P. Pringle A. (2008). Explosively launched spores of ascomycete fungi have drag-minimizing shapes. Proc. Natl. Acad. Sci. U. S. A. 105, 20583–20588. doi: 10.1073/pnas.0805017105, PMID: 19104035 PMC2634873

[ref68] Rossman A. Y. (1983). The phragmosporous species of *Nectria* and related genera. Mycol. Pap., 150, 1–164.

[ref69] Seaver F. J. (1942). Photographs and descriptions of cup-Fungi—XXXVII. Pezicula Purpurascens. Mycologia. 34, 412–415. doi: 10.1080/00275514.1942.12020911 20283549

[ref70] Shenoy B. D. Jeewon R. Wu W. P. Bhat D. J. Hyde K. D. (2006). Ribosomal and *RPB2* DNA sequence analyses suggest that *Sporidesmium* and morphologically similar genera are polyphyletic. Mycol. Res. 110, 916–928. doi: 10.1016/j.mycres.2006.06.004, PMID: 16908125

[ref71] Sherwood M. A. (1981). Convergent evolution in discomycetes from bark and wood. Bot. J. Linn. Soc. 82, 15–34. doi: 10.1111/j.1095-8339.1981.tb00948.x

[ref72] Stamatakis A. (2014). RAxML version 8: a tool for phylogenetic analysis and postanalysis of large phylogenies. Bioinformatics 30, 1312–1313. doi: 10.1093/bioinformatics/btu033, PMID: 24451623 PMC3998144

[ref73] Taşkın H. Büyükalaca S. Dogan H. H. Rehner S. A. O’Donnell K. (2010). A multigene molecular phylogenetic assessment of true morels (*Morchella*) in Turkey. Fungal Genet. Biol. 47, 672–682. doi: 10.1016/j.fgb.2010.05.004, PMID: 20580850

[ref74] Taşkın H. Büyükalaca S. Hansen K. O’Donnell K. (2012). Multilocus phylogenetic analysis of true morels (*Morchella*) reveals high levels of endemics in Turkey relative to other regions of Europe. Mycologia 104, 446–461. doi: 10.3852/11-180, PMID: 22123659

[ref75] Taşkin H. Doğan H. H. İ. Büyükalaca S. (2015). *Morchella galilaea*, an autumn species from Turkey. Mycotaxon 130, 215–221. doi: 10.5248/130.215

[ref76] Uhm J. Y. Fujii H. (1983). Heterothallism and mating type mutation in *Sclerotinia trifoliorum*. Phytopathology 73, 569–572. doi: 10.1094/Phyto-73-569

[ref77] Vaidya G. Lohman D. J. Meier R. (2011). SequenceMatrix: concatenation software for the fast assembly of multi-gene datasets with character set and codon information. Cladistics 27, 171–180. doi: 10.1111/j.1096-0031.2010.00329.x, PMID: 34875773

[ref78] Van Vooren N. (2020). Reinstatement of old taxa and publication of new genera for naming some lineages of the Pezizaceae (Ascomycota). Ascomycete Org. 12, 179–192. doi: 10.25664/art-0305

[ref79] Vinter V. Slepecky R. H. (1965). Direct transition of outgrowing bacterial spores to new sporangia without intermediate cell division. J. Bacteriol. Res. 90, 803–807. doi: 10.1128/jb.90.3.803-807.1965, PMID: 16562084 PMC315728

[ref80] Wang Z. Binder M. Hibbett D. S. (2002). A new species of *Cudonia* based on morphological and molecular data. Mycologia 94, 641–650. doi: 10.1080/15572536.2003.11833192 21156537

[ref81] White T. J. Bruns T. Lee S. Taylor J. (1990). “Amplification and direct sequencing of fungal ribosomal RNA genes for phylogenetics” in PCR protocols: A guide to methods and applications. eds. Innis M. A. Gelfand H. H. Sninsky J. J. White T. J. (San Diego: Academic Press) 315–322.

[ref82] Yao Y. J. Spooner B. M. (2000). Notes on British species of *Thecotheus* (Ascobolaceae, Pezizales), with reference to other species of the genus. Kew Bull. 55, 451–457. doi: 10.2307/4115659

[ref83] Yuan B. H. Li H. Liu L. Du X. H. (2021). Successful induction and recognition of conidiation, conidial germination and chlamydospore formation in pure culture of *Morchella*. Fungal Biol. 125, 285–293. doi: 10.1016/j.funbio.2020.11.005, PMID: 33766307

[ref84] Zeng Z. Q. Zhuang W. Y. (2016). Revision of the genus *Thyronectria* (Hypocreales) from China. Mycologia 108, 1130–1140. doi: 10.3852/16-004, PMID: 27621287

