# Supplementary material for: Cytological studies reveal high variation in ascospore number and shape and conidia produced directly from ascospores in Morchella galilaea
Source: Front Microbiol. 2023 Nov 9;14:1286501. doi: 10.3389/fmicb.2023.1286501 (PMC10690605; doi:10.3389/fmicb.2023.1286501)
Supplement: Supplementary file 1 [file Table_1.DOCX]

Supplementary Material

**Cytological studies reveal high variation in ascospore number and shape and conidia produced directly from ascospores in *Morchella galilaea***

**Xi-Hui Du*, Si-Yue Wang, Martin Ryberg, Yong-Jie Guo, Jing-Yi Wei, Donald H. Pfister****, Hanna Johannesson**

*** Correspondence:** Xi-Hui Du: [duxihuimorel@outlook.com](mailto:duxihuimorel@outlook.com)

# 1 Supplementary Tables

**Table S1** Detailed information of the retrieved sequences used for molecular phylogenetic analyses in this study.

| Species | Voucher | Locality | GenBank Accession | | | |
| --- | --- | --- | --- | --- | --- | --- |
|  |  |  | ITS | *EF1-a* | *RPB1* | *RPB2* |
| *Morchella steppicola* | M635 | Slovakia | JQ723120 | GU551543 | GU551641 | GU551690 |
| 1. *steppicola* | M512 | Hungary | JQ723119 | GU551017 | GU551100 | GU551362 |
| *M. sceptriformis* | M780 | USA | JQ723111 | GU551171 | GU551283 | GU551339 |
| *M. sceptriformis* | M887 | USA | JQ723112 | GU551187 | GU551299 | GU551355 |
| *M. americana* | M78 | USA | JQ723068 | GU551157 | GU551269 | GU551325 |
| *M. americana* | M205 | USA | JQ723078 | GU551150 | GU551262 | GU551318 |
| *M. americana* | M893 | USA | JQ723074 | GU551188 | GU551300 | GU551356 |
| *M. prava* | M38 | Canada | JQ723099 | GU551533 | GU551631 | GU551680 |
| *M. prava* | M910 | USA | JQ723100 | GU551193 | GU551305 | GU551361 |
| *M. ulmaria* | M70 | USA | JQ723089 | GU551532 | GU551630 | GU551679 |
| *M. ulmaria* | M239 | Canada | JQ723088 | GU551151 | GU551263 | GU551319 |
| *M. esculenta* | HKAS59167 | China | JQ322074 | JQ322033 | JQ322156 | JQ322197 |
| *M. esculenta* | HKAS59168 | China | JQ322075 | JQ322034 | JQ322157 | JQ322198 |
| *M. esculenta* | HKAS56676 | China | JQ322061 | JQ322020 | JQ322143 | JQ322184 |
| *M. gracilis* | M684 | Equador | JQ723086 | GU551148 | GU551260 | GU551316 |
| *M. gracilis* | M330 | Venezula | JQ723085 | GU551530 | GU551628 | GU551677 |
| *M. galilaea* | HKAS55839 | China | JQ322048 | JQ322007 | JQ322130 | JQ322171 |
| *M. galilaea* | HKAS55840 | China | JQ322049 | JQ322008 | JQ322131 | JQ322172 |
| *M. galilaea* | HAI-D-041 | Israel | JQ322065 | JQ322024 | JQ322147 | JQ322188 |
| *Morchella* sp. *Mes*-6 | HKAS56601 | China | JQ322044 | JQ322003 | JQ322126 | JQ322167 |
| *Morchella* sp. *Mes*-6 | HKAS59163 | China | JQ322073 | JQ322032 | JQ322155 | JQ322196 |
| *Morchella* sp. *Mes*-9 | HKAS59118 | China | JQ322066 | JQ322025 | JQ322148 | JQ322189 |
| *Morchella* sp. *Mes*-9 | HKAS59121 | China | JQ322067 | JQ322026 | JQ322149 | JQ322190 |
| *Morchella* sp. *Mes*-9 | HKAS59123 | China | JQ322068 | JQ322027 | JQ322150 | JQ322191 |
| *Morchella* sp. *Mes*-15 | HKAS62913 | China | JQ322046 | JQ322005 | JQ322128 | JQ322169 |
| *Morchella* sp. *Mes*-15 | HKAS62914 | China | JQ322047 | JQ322006 | JQ322129 | JQ322170 |
| *Morchella* sp. *Mes*-25 | HKAS62861 | China | JQ322076 | JQ322035 | JQ322158 | JQ322199 |
| *Morchella* sp. *Mes*-25 | HKAS62862 | China | JQ322077 | JQ322036 | JQ322159 | JQ322200 |
| *Morchella* sp. *Mes*-26 | HKAS55912 | China | JQ322055 | JQ322014 | JQ322137 | JQ322178 |
| *Morchella* sp. *Mes*-26 | HKAS55913 | China | JQ322056 | JQ322015 | JQ322138 | JQ322179 |

**Table S2** Ascospore and ascus size of one-spored to 16-spored asci of *Morchella galiaea* and the corresponding specimens.

| Number of spores in asci | Ascospore | | Ascus | | Specimen |
| --- | --- | --- | --- | --- | --- |
|  | Length (μm) | Width (μm) | Length (μm) | Width (μm) |  |
| 1 | 16.03 | 8.81 | 260.21 | 22.96 | FCNU1118 |
| 2 | 16.40 | 12.95 | 268.77 | 22.51 | FCNU1117 |
|  | 14.58 | 10.54 |  |  |  |
| 2 | 15.51 | 9.36 | 309.79 | 16.70 | FCNU1118 |
|  | 16.25 | 9.41 |  |  |  |
| 2 | 29.13 | 8.99 | 364.36 | 21.55 | FCNU1118 |
|  | 25.15 | 10.89 |  |  |  |
| 2 | 18.10 | 8.70 | 285.02 | 16.47 | FCNU1118 |
|  | 17.60 | 9.14 |  |  |  |
| 3 | 20.65 | 5.93 | 280.81 | 17.93 | FCNU1118 |
|  | 15.01 | 7.66 |  |  |  |
|  | 21.04 | 9.09 |  |  |  |
| 3 | 23.11 | 7.29 | 336.49 | 17.90 | FCNU1118 |
|  | 17.01 | 8.11 |  |  |  |
|  | 24.20 | 7.99 |  |  |  |
| 4 | 15.68 | 10.64 | 308.24 | 22.48 | FCNU1118 |
|  | 13.29 | 9.82 |  |  |  |
|  | 22.20 | 8.00 |  |  |  |
|  | 25.03 | 11.04 |  |  |  |
| 4 | 10.76 | 8.31 | 232.63 | 17.22 | FCNU1118 |
|  | 10.59 | 7.36 |  |  |  |
|  | 14.00 | 6.54 |  |  |  |
|  | 8.52 | 6.57 |  |  |  |
| 5 | 15.78 | 8.11 | 302.69 | 20.14 | FCNU1118 |
|  | 14.41 | 8.02 |  |  |  |
|  | 16.17 | 9.88 |  |  |  |
|  | 16.89 | 8.67 |  |  |  |
|  | 19.26 | 9.73 |  |  |  |
| 5 | 18.63 | 9.69 | 291.06 | 20.46 | FCNU1118 |
|  | 14.69 | 9.20 |  |  |  |
|  | 16.54 | 9.27 |  |  |  |
|  | 15.82 | 10.03 |  |  |  |
|  | 16.09 | 9.00 |  |  |  |
| 5 | 17.38 | 11.24 | 289.79 | 22.22 | FCNU1061 |
|  | 17.86 | 11.33 |  |  |  |
|  | 16.07 | 11.19 |  |  |  |
|  | 19.11 | 9.98 |  |  |  |
|  | 20.07 | 10.03 |  |  |  |
| 5 | 11.95 | 8.58 | 305.56 | 17.80 | FCNU1119 |
|  | 15.02 | 13.38 |  |  |  |
|  | 15.15 | 14.06 |  |  |  |
|  | 13.83 | 12.05 |  |  |  |
|  | 11.95 | 8.97 |  |  |  |
| 6 | 22.76 | 10.85 | 320.13 | 21.14 | FCNU1116 |
|  | 27.33 | 9.69 |  |  |  |
|  | 21.47 | 8.23 |  |  |  |
|  | 19.76 | 9.71 |  |  |  |
|  | 20.93 | 8.74 |  |  |  |
|  | 14.20 | 8.49 |  |  |  |
| 6 | 25.52 | 8.78 | 334.61 | 20.19 | FCNU1118 |
|  | 27.32 | 8.49 |  |  |  |
|  | 24.20 | 9.13 |  |  |  |
|  | 23.75 | 9.83 |  |  |  |
|  | 22.16 | 9.11 |  |  |  |
|  | 24.07 | 9.31 |  |  |  |
| 6 | 21.33 | 10.54 | 287.80 | 20.87 | FCNU1118 |
|  | 19.26 | 10.54 |  |  |  |
|  | 17.44 | 12.36 |  |  |  |
|  | 15.78 | 12.56 |  |  |  |
|  | 18.40 | 12.40 |  |  |  |
|  | 18.73 | 12.78 |  |  |  |
| 6 | 19.31 | 9.51 | 275.68 | 18.52 | FCNU1118 |
|  | 22.78 | 8.10 |  |  |  |
|  | 17.64 | 9.53 |  |  |  |
|  | 18.22 | 9.82 |  |  |  |
|  | 19.63 | 9.82 |  |  |  |
|  | 25.59 | 8.93 |  |  |  |
| 7 | 8.23 | 8.01 | 215.60 | 20.04 | FCNU1116 |
|  | 9.32 | 8.69 |  |  |  |
|  | 9.46 | 8.66 |  |  |  |
|  | 8.60 | 7.37 |  |  |  |
|  | 9.39 | 8.58 |  |  |  |
|  | 9.40 | 9.20 |  |  |  |
|  | 9.76 | 9.27 |  |  |  |
| 7 | 20.36 | 9.36 | 298.65 | 20.20 | FCNU1118 |
|  | 21.64 | 10.33 |  |  |  |
|  | 22.81 | 10.08 |  |  |  |
|  | 19.78 | 10.28 |  |  |  |
|  | 22.15 | 9.59 |  |  |  |
|  | 24.31 | 9.98 |  |  |  |
|  | 21.11 | 8.23 |  |  |  |
| 7 | 19.38 | 7.70 | 279.66 | 22.74 | FCNU1118 |
|  | 21.97 | 8.48 |  |  |  |
|  | 19.61 | 9.80 |  |  |  |
|  | 20.95 | 9.18 |  |  |  |
|  | 12.27 | 7.91 |  |  |  |
|  | 22.44 | 10.14 |  |  |  |
|  | 24.45 | 10.52 |  |  |  |
| 7 | 20.98 | 8.23 | 287.58 | 20.44 | FCNU1118 |
|  | 23.24 | 8.17 |  |  |  |
|  | 20.11 | 9.60 |  |  |  |
|  | 20.02 | 9.37 |  |  |  |
|  | 20.54 | 9.23 |  |  |  |
|  | 18.58 | 8.17 |  |  |  |
|  | 20.57 | 9.06 |  |  |  |
| 8 | 19.62 | 11.20 | 290.93 | 22.74 | FCNU1118 |
|  | 18.91 | 12.59 |  |  |  |
|  | 19.17 | 12.33 |  |  |  |
|  | 18.56 | 12.13 |  |  |  |
|  | 19.78 | 12.27 |  |  |  |
|  | 18.77 | 12.13 |  |  |  |
|  | 19.50 | 12.50 |  |  |  |
|  | 20.24 | 12.23 |  |  |  |
| 8 | 20.77 | 10.96 | 310.61 | 17.68 | FCNU1118 |
|  | 20.50 | 11.27 |  |  |  |
|  | 21.73 | 11.20 |  |  |  |
|  | 21.47 | 11.52 |  |  |  |
|  | 19.56 | 10.70 |  |  |  |
|  | 19.61 | 19.61 |  |  |  |
|  | 20.83 | 11.30 |  |  |  |
|  | 21.98 | 10.80 |  |  |  |
| 8 | 22.12 | 14.49 | 314.09 | 23.87 | FCNU1118 |
|  | 22.44 | 15.17 |  |  |  |
|  | 22.55 | 14.98 |  |  |  |
|  | 22.79 | 15.16 |  |  |  |
|  | 22.88 | 14.85 |  |  |  |
|  | 22.90 | 14.70 |  |  |  |
|  | 23.35 | 14.03 |  |  |  |
|  | 23.39 | 14.49 |  |  |  |
| 8 | 22.88 | 11.05 | 294.14 | 23.49 | FCNU1061 |
|  | 22.88 | 11.02 |  |  |  |
|  | 20.09 | 9.37 |  |  |  |
|  | 20.36 | 11.55 |  |  |  |
|  | 20.12 | 11.75 |  |  |  |
|  | 19.85 | 11.62 |  |  |  |
|  | 21.63 | 10.33 |  |  |  |
|  | 21.85 | 10.45 |  |  |  |
| 9 | 17.82 | 11.65 | 283.93 | 22.61 | FCNU1118 |
|  | 18.71 | 12.77 |  |  |  |
|  | 17.35 | 12.61 |  |  |  |
|  | 8.66 | 7.65 |  |  |  |
|  | 9.46 | 7.51 |  |  |  |
|  | 19.35 | 12.92 |  |  |  |
|  | 19.84 | 9.41 |  |  |  |
|  | 18.70 | 11.91 |  |  |  |
|  | 26.16 | 11.20 |  |  |  |
| 9 | 14.07 | 8.73 | 318.62 | 21.50 | FCNU1119 |
|  | 18.76 | 8.63 |  |  |  |
|  | 19.06 | 9.86 |  |  |  |
|  | 19.21 | 9.41 |  |  |  |
|  | 19.23 | 10.08 |  |  |  |
|  | 19.72 | 8.91 |  |  |  |
|  | 19.86 | 9.90 |  |  |  |
|  | 20.39 | 9.21 |  |  |  |
|  | 20.74 | 8.34 |  |  |  |
| 10 | 16.25 | 9.49 | 339.53 | 18.27 | FCNU1118 |
|  | 16.47 | 8.29 |  |  |  |
|  | 16.65 | 10.29 |  |  |  |
|  | 17.25 | 10.26 |  |  |  |
|  | 17.31 | 10.92 |  |  |  |
|  | 17.32 | 7.68 |  |  |  |
|  | 18.46 | 9.49 |  |  |  |
|  | 18.75 | 10.64 |  |  |  |
|  | 18.96 | 11.35 |  |  |  |
|  | 19.29 | 10.88 |  |  |  |
| 11 | 11.57 | 8.13 | /^*^ | / | FCNU1117 |
|  | 11.94 | 7.65 |  |  |  |
|  | 12.23 | 7.68 |  |  |  |
|  | 12.68 | 6.75 |  |  |  |
|  | 13.49 | 7.65 |  |  |  |
|  | 13.77 | 7.01 |  |  |  |
|  | 13.77 | 7.05 |  |  |  |
|  | 15.18 | 7.49 |  |  |  |
|  | 15.26 | 7.46 |  |  |  |
|  | 16.96 | 8.40 |  |  |  |
|  | 20.86 | 11.44 |  |  |  |
| 12 | 13.15 | 6.78 | 317.96 | 22.78 | FCNU1118 |
|  | 13.18 | 6.82 |  |  |  |
|  | 14.46 | 7.71 |  |  |  |
|  | 15.07 | 10.18 |  |  |  |
|  | 15.60 | 7.65 |  |  |  |
|  | 15.68 | 11.14 |  |  |  |
|  | 16.47 | 10.92 |  |  |  |
|  | 16.72 | 12.13 |  |  |  |
|  | 16.85 | 11.01 |  |  |  |
|  | 16.90 | 10.11 |  |  |  |
|  | 17.05 | 9.69 |  |  |  |
|  | 17.51 | 11.13 |  |  |  |
| 13 | 11.91 | 9.25 | / | / | FCNU1118 |
|  | 15.58 | 7.56 |  |  |  |
|  | 11.87 | 8.45 |  |  |  |
|  | 13.07 | 7.59 |  |  |  |
|  | 15.42 | 9.49 |  |  |  |
|  | 13.88 | 9.88 |  |  |  |
|  | 11.55 | 8.95 |  |  |  |
|  | 10.82 | 8.56 |  |  |  |
|  | 20.86 | 10.25 |  |  |  |
|  | 12.13 | 7.13 |  |  |  |
|  | 13.80 | 9.31 |  |  |  |
|  | 12.15 | 8.99 |  |  |  |
|  | 13.58 | 6.65 |  |  |  |
| 14 | 7.84 | 6.03 | 286.80 | 21.37 | FCNU1118 |
|  | 11.64 | 6.01 |  |  |  |
|  | 12.15 | 6.86 |  |  |  |
|  | 12.57 | 6.99 |  |  |  |
|  | 12.85 | 7.84 |  |  |  |
|  | 13.46 | 7.88 |  |  |  |
|  | 15.39 | 10.42 |  |  |  |
|  | 15.60 | 9.65 |  |  |  |
|  | 15.69 | 10.55 |  |  |  |
|  | 16.30 | 10.93 |  |  |  |
|  | 16.48 | 10.46 |  |  |  |
|  | 17.33 | 10.46 |  |  |  |
|  | 17.73 | 11.88 |  |  |  |
|  | 18.03 | 10.16 |  |  |  |
| 15 | 11.66 | 7.25 | 273.56 | 23.32 | FCNU1118 |
|  | 13.43 | 7.50 |  |  |  |
|  | 13.39 | 7.28 |  |  |  |
|  | 13.42 | 7.51 |  |  |  |
|  | 11.47 | 6.51 |  |  |  |
|  | 12.86 | 6.12 |  |  |  |
|  | 12.51 | 7.43 |  |  |  |
|  | 15.25 | 7.73 |  |  |  |
|  | 14.69 | 8.01 |  |  |  |
|  | 12.95 | 7.95 |  |  |  |
|  | 14.77 | 7.85 |  |  |  |
|  | 12.29 | 7.29 |  |  |  |
|  | 14.27 | 7.77 |  |  |  |
|  | 10.27 | 7.06 |  |  |  |
|  | 15.91 | 8.18 |  |  |  |
| 16 | 8.75 | 7.57 | / | / | FCNU1118 |
|  | 10.04 | 6.89 |  |  |  |
|  | 10.66 | 8.07 |  |  |  |
|  | 10.76 | 6.75 |  |  |  |
|  | 11.46 | 8.10 |  |  |  |
|  | 11.87 | 5.82 |  |  |  |
|  | 11.96 | 7.98 |  |  |  |
|  | 12.15 | 8.78 |  |  |  |
|  | 12.47 | 6.49 |  |  |  |
|  | 12.96 | 9.02 |  |  |  |
|  | 13.70 | 9.03 |  |  |  |
|  | 13.52 | 8.89 |  |  |  |
|  | 13.72 | 8.20 |  |  |  |
|  | 14.08 | 6.76 |  |  |  |
|  | 14.08 | 8.66 |  |  |  |
|  | 14.79 | 6.78 |  |  |  |

* The ascus was broken and incomplete, so no data was provided for its length and width .

**Table S3** Number of different-spored asci from 654 asci of *Morchella galiaea* in this study.

| Different-spored asci | Ascus number observed from 654 asci | Specimens |
| --- | --- | --- |
| Unispored ascus | 2 | FCNU1117, FCNU1118 |
| Two-spored ascus | 7 | FCNU1117, FCNU1118, FCNU1119 |
| Three-spored ascus | 5 | FCNU1116, FCNU1118 |
| Four-spored ascus | 13 | FCNU1116, FCNU1118 |
| Five-spored ascus | 13 | FCNU1061, FCNU1116, FCNU1118, FCNU1119 |
| Six-spored ascus | 11 | FCNU1116, FCNU1117, FCNU1118, FCNU1119 |
| Seven-spored ascus | 17 | FCNU1116, FCNU1117, FCNU1118, FCNU1119 |
| Eight-spored ascus | 540 | FCNU1061, FCNU1116, FCNU1117, FCNU1118, FCNU1119 |
| Nine-spored ascus | 17 | FCNU1061, FCNU1116, FCNU1117, FCNU1118, FCNU1119 |
| Ten-spored ascus | 12 | FCNU1061, FCNU1117, FCNU1118 |
| 11-spored ascus | 6 | FCNU1117, FCNU1118 |
| 12-spored ascus | 3 | FCNU1118 |
| 13-spored ascus | 3 | FCNU1118 |
| 14-spored ascus | 2 | FCNU1118 |
| 15-spored ascus | 2 | FCNU1118 |
| 16-spored ascus | 1 | FCNU1118 |
